# One-dimensional architectures in polymer solid electrolytes: design principles, ion-transport mechanisms and practical implementation

**DOI:** 10.1039/d6sc05793d

**Published:** 2026-09-14

**Authors:** Tian Wang, Xinyang Li, Dan He, Shujiang Ding, Na Li

**Affiliations:** a School of Chemistry, Engineering Research Center of Energy Storage Materials and Devices, Ministry of Education, National Innovation Platform (Center) for Industry-Education Integration of Energy Storage Technology, Xi'an Jiaotong University Xi'an 710049 China lina0831@xjtu.edu.cn dingsj@xjtu.edu.cn

## Abstract

Polymer solid electrolytes (PSEs) are promising for practical solid-state batteries due to their interfacial adaptability, film-forming ability, and processability. However, they still suffer from low ionic conductivity, low Li^+^ transference number, and insufficient mechanical support, which originate from disordered Li^+^ pathways, inadequate suppression of anion migration, and weak load-bearing frameworks. One-dimensional (1D) architectures offer a structural design paradigm to address these interconnected limitations by engineering oriented Li^+^ transport pathways, long-range functional interfaces, and percolated supporting networks. This review systematically examines 1D architectures in PSEs, first summarizing their classification, critical design variables, and fabrication methods. Mechanistically, it focuses on how oriented structures reduce Li^+^ transport tortuosity, continuous interfaces promote interfacial Li^+^ transport and amplify interfacial regulation, and interconnected supporting skeletons enhance stress transfer and mechanical integrity. This review further discusses the remaining challenges in this field, including standardization of structural descriptors, mechanistic decoupling, high-voltage stability evaluation, scalable fabrication, and validation under realistic battery conditions. Overall, 1D architectures are not only structural units in PSEs, but also a design paradigm for the coordinated optimization of Li^+^ transport pathways, interfacial functions, and mechanical stability, providing new insights into the construction of high-performance solid-state electrolytes.

## Abstract 2

1D architectures in polymer electrolytes regulate Li^+^ transport, interfacial functions, and stress through spatial continuity and directionality, enabling pathway-level design for practical solid-state batteries.

## Graphical abstract

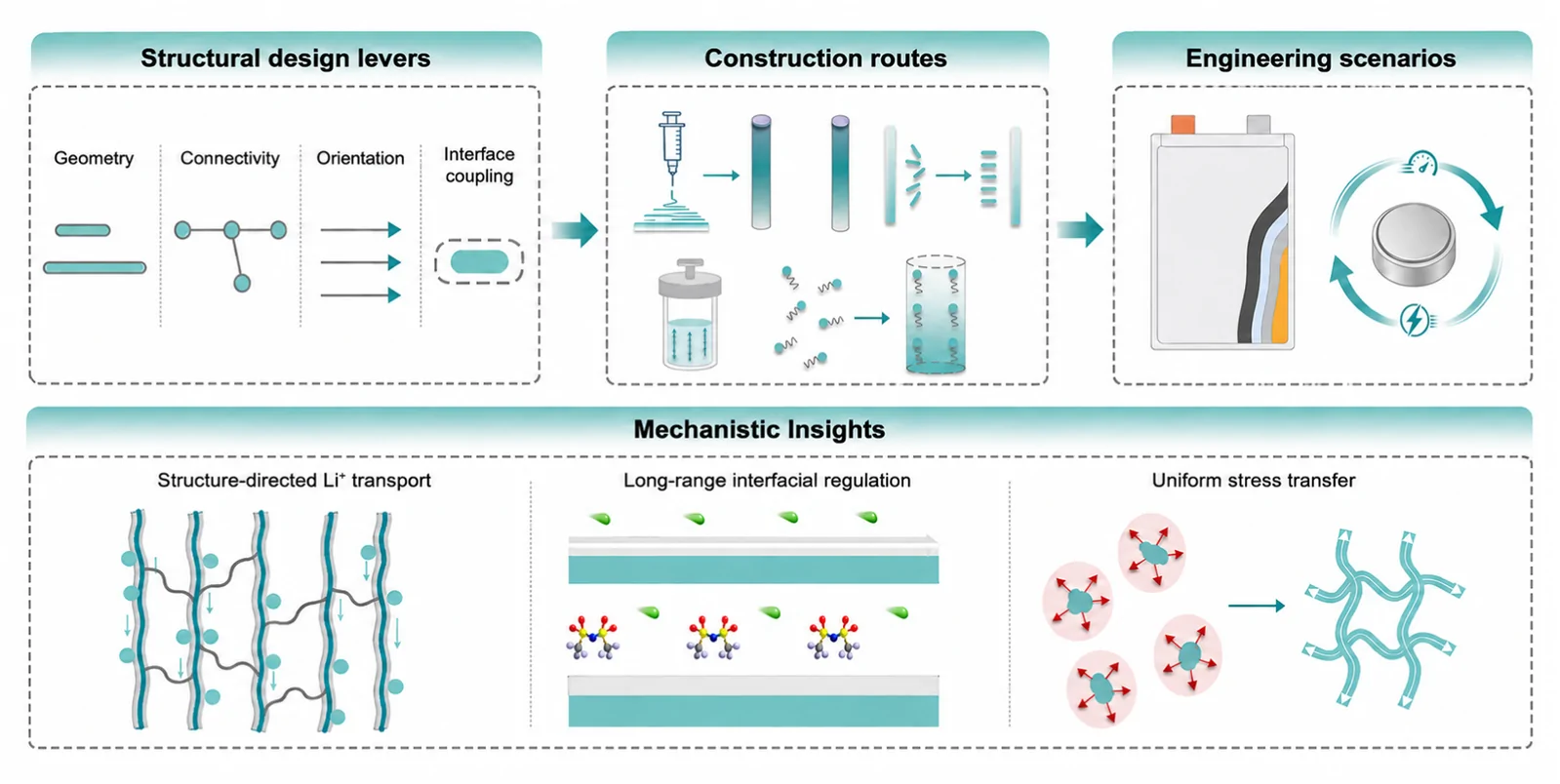

## 1 Introduction

The growing demand for electric vehicles and grid-scale energy storage has accelerated the development of solid-state lithium batteries with higher safety and energy density.^1–6^ Among solid electrolytes, inorganic solid electrolytes (ISEs) generally offer high ionic conductivity but are often limited by rigid interfacial contact, air sensitivity, and unstable electrode/electrolyte compatibility.^7–13^ In contrast, polymer solid electrolytes (PSEs) provide better interfacial compliance, film-forming ability, flexibility, and scalable processability.^14–17^ However, their practical application remains constrained by sluggish Li^+^ transport and insufficient mechanical robustness.^18–20^ In many PSEs, Li^+^ migration is strongly coupled with polymer segmental motion, while mobile anions and heterogeneous interfaces intensify concentration polarization and nonuniform ion flux.^21–23^

To overcome these bottlenecks, several strategies have been developed.^24^ Polymer matrix design is the most direct route.^25^ Through copolymerization, crosslinking, grafting, or flexible chain engineering, polymer crystallization and segmental dynamics can be effectively regulated.^26,27^ Solvation regulation provides another molecular-level strategy.^28^ By optimizing lithium salts and Li^+^ coordination environments, it can increase the concentration of mobile Li^+^ and suppress anion migration.^29,30^ Inorganic fillers further extend the design space beyond the polymer matrix itself.^31^ Inert fillers modulate polymer chains and lithium salts through surface interactions, whereas active fillers provide additional Li^+^ migration sites, together with enhanced thermal stability and mechanical reinforcement.^32,33^

However, conventional zero-dimensional (0D) particle fillers are intrinsically constrained by their geometry.^34^ As spatially isolated units, they force Li^+^ to migrate repeatedly through the polymer phase and filler interfaces. At low particle contents, interfacial regions are difficult to connect into continuous channels. At excessive loadings, fillers tend to aggregate and disrupt the continuity of the polymer phase.^35^ Consequently, 0D particle fillers mainly improve the local transport environment but rarely establish stable, electrolyte-spanning Li^+^ migration pathways.

For this reason, the geometry and spatial distribution of fillers are critical. One-dimensional (1D) nanowires, nanofibers, and nanotubes possess high aspect ratios and are therefore more likely to contact each other than their 0D counterparts. They can form continuous or quasi-continuous networks at much lower volume fractions.^36^ In these configurations, interfacial effects generated on filler surfaces can propagate along the axial direction rather than remaining confined around isolated particles. This enables long-range functional connectivity across the electrolyte. More importantly, 1D architectures should not be regarded merely as an alternative filler morphology but as a structural design strategy.

**Fig. 1:**
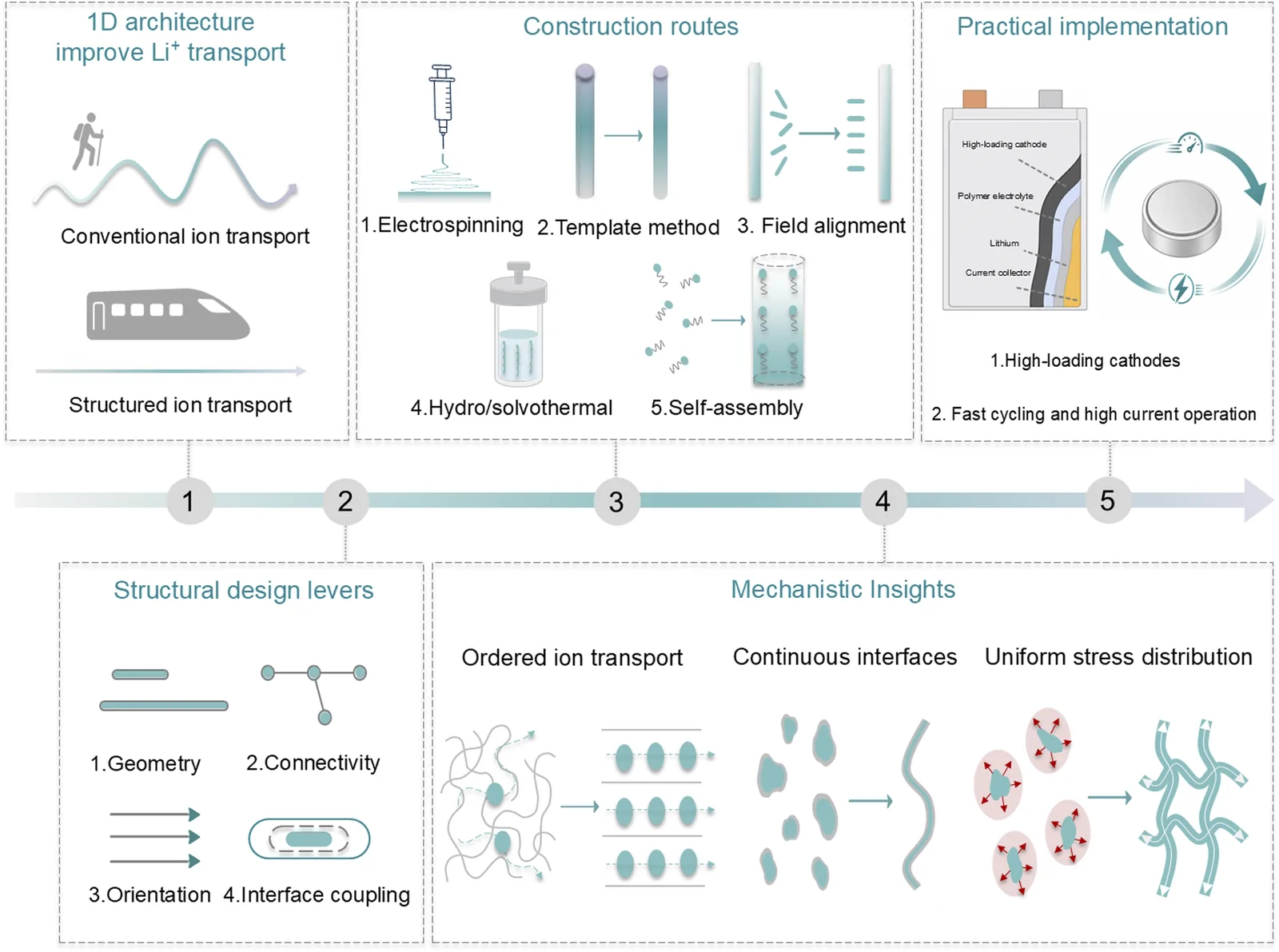
Schematic illustration of the scope of this review.

In this review, we systematically examine the design principles, ion-transport mechanisms, and engineering scenarios of 1D architectures in polymer electrolytes for solid-state batteries (Fig. 1). We first define 1D architectures and classify them by Li^+^-conducting capability and spatial organization. We then discuss key design variables, including geometric dimensions, spatial continuity, orientation degree, and interfacial configuration, followed by major construction methods such as electrospinning, template synthesis, external-field induction, hydrothermal/solvothermal approaches, and molecular self-assembly. We then elucidate the three governing regulation mechanisms through which 1D architectures enhance electrolyte performance: structure-directed directional Li^+^ transport, long-range interfacial regulation, and long-range stress transfer. The practical viability of polymer electrolytes incorporating 1D architectures is subsequently evaluated under demanding operating conditions, specifically high cathode mass loading, elevated charge–discharge rates, and high current densities. Finally, we identify five critical challenges that must be addressed: the lack of unified structural evaluation standards, the difficulty of isolating the intrinsic contributions of 1D architectures, the poorly understood role of 1D architectures in high-voltage interfacial stability, the imperative of demonstrating manufacturing scalability, and the necessity of rigorous validation under realistic battery operating conditions.

## 2 Classification and structural design of 1D architectures

### 2.1 Concept of 1D architectures

In this review, 1D architectures are defined as structural units with directional continuity in one dimension. This concept includes conventional 1D materials, such as nanowires, nanorods, nanofibers, and nanotubes.^37^ It also includes derived structures based on these units, including hollow structures, core–shell structures, hierarchical structures, heterostructures, and surface-functionalized structures. In addition, interconnected fibrous networks, aligned arrays, 1D channels constructed by vertically organized particles or nanosheets, and molecular or supramolecular ion-conducting channels formed through self-assembly are also included in the scope of this review. Therefore, 1D architectures are not defined only by the intrinsic morphology of the materials. More importantly, they should be assessed by whether they can construct Li^+^ migration pathways, extend functional interfaces, and provide mechanical support in polymer electrolytes. Thus, 1D architectures should not be regarded as a single morphological concept. They should instead be understood as a structural design strategy. This strategy uses spatial continuity and directionality to convert local functions into continuous and directional functions across the electrolyte. To keep this broad scope from admitting any anisotropic feature, a 1D architecture is defined here. A structure qualifies when it meets at least one of the following conditions: (i) it possesses an aspect ratio high enough to favor directional continuity rather than isolated point contact; (ii) it establishes a continuous or quasi-continuous Li^+^-conducting pathway or functional interface that spans the electrolyte; (iii) it exhibits a measurable transport anisotropy, such as anisotropic ^7^Li diffusion or direction-dependent conductivity.

### 2.2 Classification of 1D architectures

#### 2.2.1 Classification by intrinsic Li^+^ conductivity

1D architectures can be classified into intrinsically Li^+^-conductive and non-intrinsically Li^+^-conductive types. Li^+^-conductive ceramic nanowires, such as lithium lanthanum zirconium oxide (LLZO),^38^ lithium lanthanum titanate (LLTO),^39^ and lithium aluminum titanium phosphate (LATP),^40^ directly provide Li^+^ migration sites. In contrast, fillers such as SiO_2_,^41^ CeO_2_,^42^ Al_2_O_3_,^43^ and cellulose nanofibers,^44^ mainly rely on interfacial Lewis acidic and basic sites and polar functional groups to promote lithium salt dissociation, immobilize anions, and improve mechanical properties.

#### 2.2.2 Classification by spatial organization

From the perspective of spatial organization, 1D architectures in polymer electrolytes can be classified into randomly dispersed structures, continuous networks, and aligned structures.

##### 2.2.2.1 Randomly dispersed 1D architectures

**Fig. 2:**
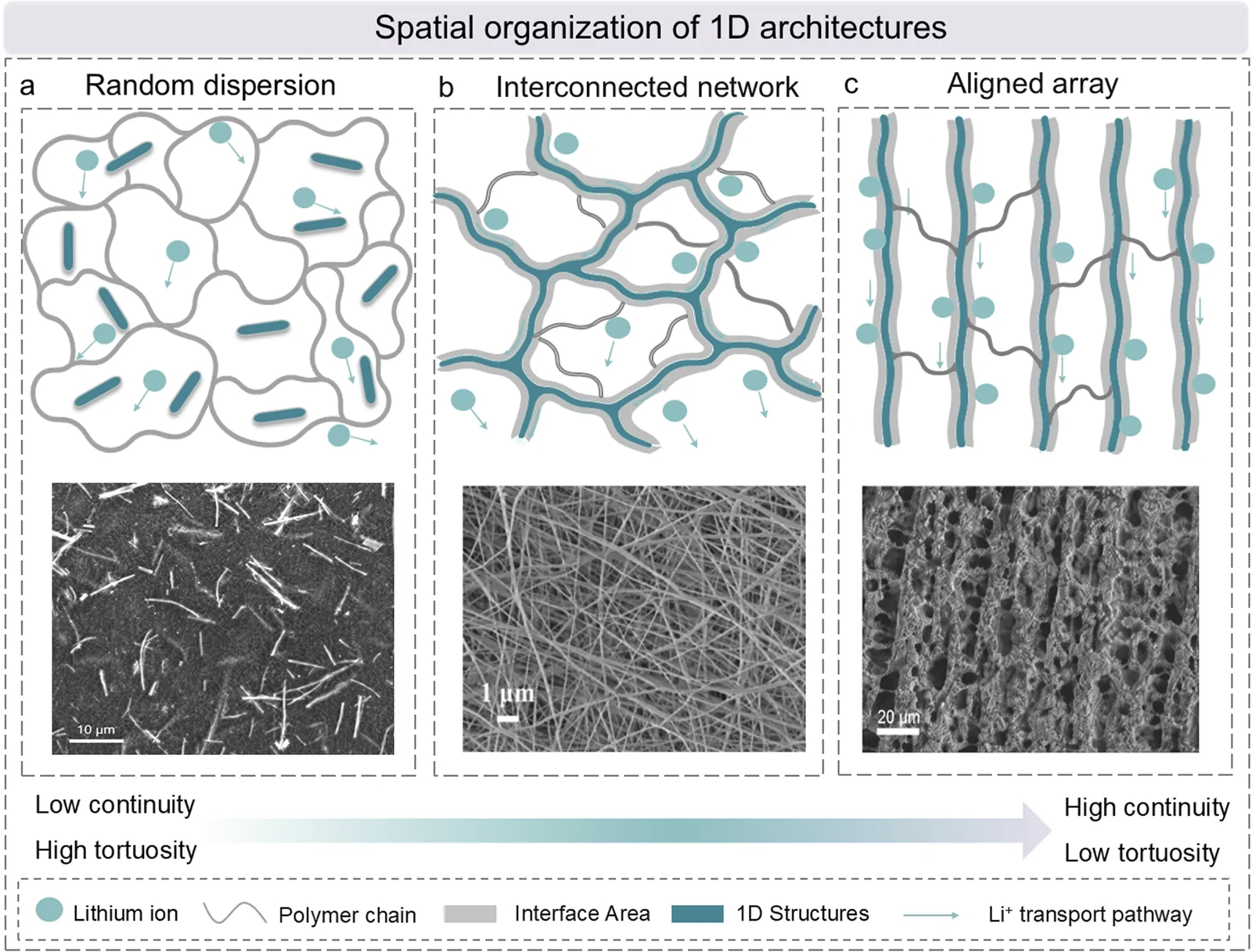
Spatial organization of 1D architectures in polymer electrolytes. (a) Randomly dispersed 1D units. Adapted with permission.^47^ Copyright 2015, American Chemical Society. (b) Interconnected 1D networks. Adapted with permission.^48^ Copyright 2025, American Chemical Society. (c) Aligned 1D arrays. Adapted with permission.^50^ Copyright 2017, American Chemical Society.

Randomly dispersed 1D architectures are the most common form of 1D organization in polymer electrolytes (Fig. 2a). In this type, nanowires, nanofibers, nanotubes, or nanorods are distributed in the polymer matrix without preferred orientation. For example, Lin *et al.*^45^ composited halloysite nanotubes with PEO. Wang *et al.*^46^ incorporated hydroxyapatite nanowires into PEO electrolyte. Liu *et al.*^47^ introduced LLTO nanowires into PAN electrolyte. In these cases, the randomly dispersed 1D fillers increased filler polymer contact and extended local interfacial regions, but they did not necessarily form continuous or directional Li^+^ transport pathways.

##### 2.2.2.2 Continuously connected 1D networks

Continuously connected 1D networks are formed when individual 1D units overlap, interweave, or connect with each other in the electrolyte (Fig. 2b). For example, Liu *et al.*^48^ constructed PEO electrolyte containing calcium alginate (CA) nanofiber network. Song *et al.*^49^ fabricated PVDF/PEO fibrous membranes *via* electrospinning. These structures provided more continuous transport interfaces and supporting skeletons than randomly dispersed 1D fillers.

##### 2.2.2.3 Aligned 1D architectures

In this type, 1D units or channels are arranged along a target direction, such as the through-thickness or in-plane direction of the electrolyte (Fig. 2c). This ordered arrangement can reduce Li^+^ transport tortuosity and promote anisotropic ion migration. For example, Zhai *et al.*^50^ formed 1D channels by vertically aligning LATP nanoparticles in PEO matrix, leading to an ionic conductivity 3.6 times that of the randomly dispersed LATP counterpart. Wang *et al.*^51^ immobilized lithium montmorillonite (Li-MMT) nanosheets within vertically aligned PVDF-HFP channels. In both cases, the ion transport structures were arranged along a target direction. These aligned architectures reduced transport tortuosity and promoted directional Li^+^ migration.

### 2.3 Design variables of 1D architectures

1D architectures should not be regarded as rigid structural categories, but as flexible design frameworks.^52^ The ion migration behavior in polymer electrolytes can be regulated by tuning the geometry and morphology, spatial continuity, orientation, and interfacial configuration of 1D architectures.

**Fig. 3:**
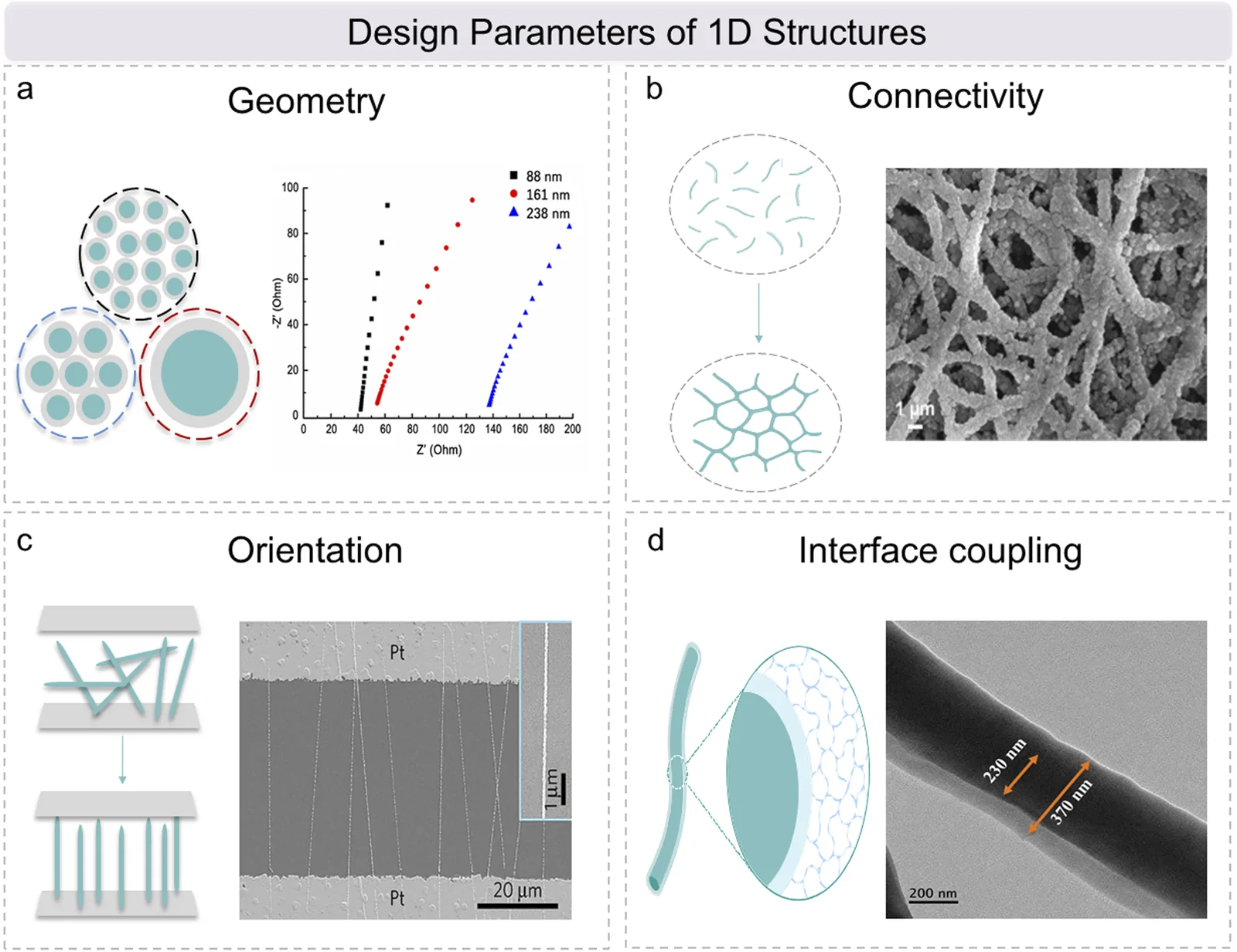
Structural design parameters of 1D architectures in polymer electrolytes. (a) Diameter-controlled LLTO nanowires. Adapted with permission.^53^ Copyright 2022, Elsevier. (b) Interconnected ZIF-8@PI fiber network. Adapted with permission.^56^ Copyright 2022, Wiley-VCH. (c) Aligned LLTO nanowire pathways. Adapted with permission.^57^ Copyright 2017, Springer Nature. (d) PDA–PEI coupled LLTO interface. Adapted with permission.^59^ Copyright 2024, Elsevier.

Geometry and morphology mainly determine the contact area and interfacial coupling between 1D units and the polymer matrix. At a given filler content, smaller diameters generally provide larger surface areas and more organic–inorganic interfaces (Fig. 3a). For example, Nourisabet *et al.*^53^ prepared PEO/PVDF composite electrolytes containing LLTO nanowires with diameters of 88, 161, and 238 nm. The electrolyte containing 88 nm nanowires showed the highest ionic conductivity of 6.02 × 10^−3^ S cm^−1^, compared with 4.17 × 10^−3^ S cm^−1^ for 161 nm nanowires and 2.04 × 10^−3^ S cm^−1^ for 238 nm nanowires. In addition to diameter, morphology is another factor that influences interfacial contact and coupling. Li *et al.*^54^ combined zinc oxide (ZnO) hollow nanotubes with PEO–lithium bis(trifluoromethanesulfonyl)imide (LiTFSI) electrolyte. The hollow nanotubes increased the filler/polymer contact area through their inner and outer interfaces and extended the interfacial region. Hu *et al.*^55^ used gradient electrospinning to prepare hierarchical LLTO nanotube fillers for PAN-based polymer electrolytes. Unlike conventional nanowires, these nanotubes are composed of interconnected small particles. The resulting porous channels promote polymer infiltration and improve coupling with the polymer matrix.

Spatial continuity is key to converting local filler functions into electrolyte-level transport benefits. Du *et al.*^56^ embedded 1D metal–organic framework (MOF) fibers assembled from zeolitic imidazolate framework-8 (ZIF-8) nanocrystals on polyimide (PI) fibers into PVDF matrix. This structure provided continuous interfaces for regulating ion transport on the micrometer scale (Fig. 3b). Compared with randomly dispersed MOF particles, this structure increased the room-temperature ionic conductivity from 8.55 × 10^−5^ to 4.08 × 10^−4^ S cm^−1^ and reduced the activation energy from 0.18 to 0.10 eV.

Directional alignment is critical for reducing the tortuosity of ion transport pathways. For example, aligned LLTO nanowires create continuous, junction-free Li^+^ conduction pathways along their surfaces. Liu *et al.*^57^ combined aligned LLTO nanowires with PAN-LiClO_4_ electrolyte (Fig. 3c). The resulting electrolyte exhibited room-temperature ionic conductivity of 6.05 × 10^−5^ S cm^−1^, whereas the randomly dispersed nanowire counterpart showed only 5.40 × 10^−6^ S cm^−1^. Lan *et al.*^58^ further showed that orientation design can also transform anisotropic ion conductors into continuous through-thickness pathways. By aligning LiMPS nanosheets perpendicular to the electrodes, the PA-LiMPS/PEO electrolyte converts the intrinsic in-plane superionic conduction of LiMPS into effective Li^+^ transport across the electrolyte. This pathway-level alignment avoids tortuous conduction in randomly distributed nanosheets and gives the composite polymer electrolyte a high room-temperature ionic conductivity of 10.2 mS cm^−1^.

Different interfacial configurations allow 1D architectures to regulate ion transport in different ways. Lee *et al.*^59^ functionalized LLTO nanowires with polydopamine–polyethyleneimine (PDA–PEI), where amino and imine groups helped immobilize free anions from the lithium salt and increase the Li^+^ transference number (Fig. 3d). Cheng *et al.*^60^ introduced PEA-coated inorganic cluster chains into PVDF-HFP matrix. Hydrogen bonding between PEA and PVDF-HFP not only induced the formation of the β phase in PVDF-HFP, but also improved the compatibility of the cluster chains with the PVDF-HFP matrix.

Therefore, the design of 1D architectures should emphasize the coordinated regulation of geometry and morphology, spatial continuity, orientation, and interfacial configuration, rather than any single structural parameter. Only through such synergy can 1D units effectively build continuous transport pathways, optimize interfacial interactions, and regulate Li^+^ migration at the electrolyte level.

## 3 Design methods for 1D architectures

**Fig. 4:**
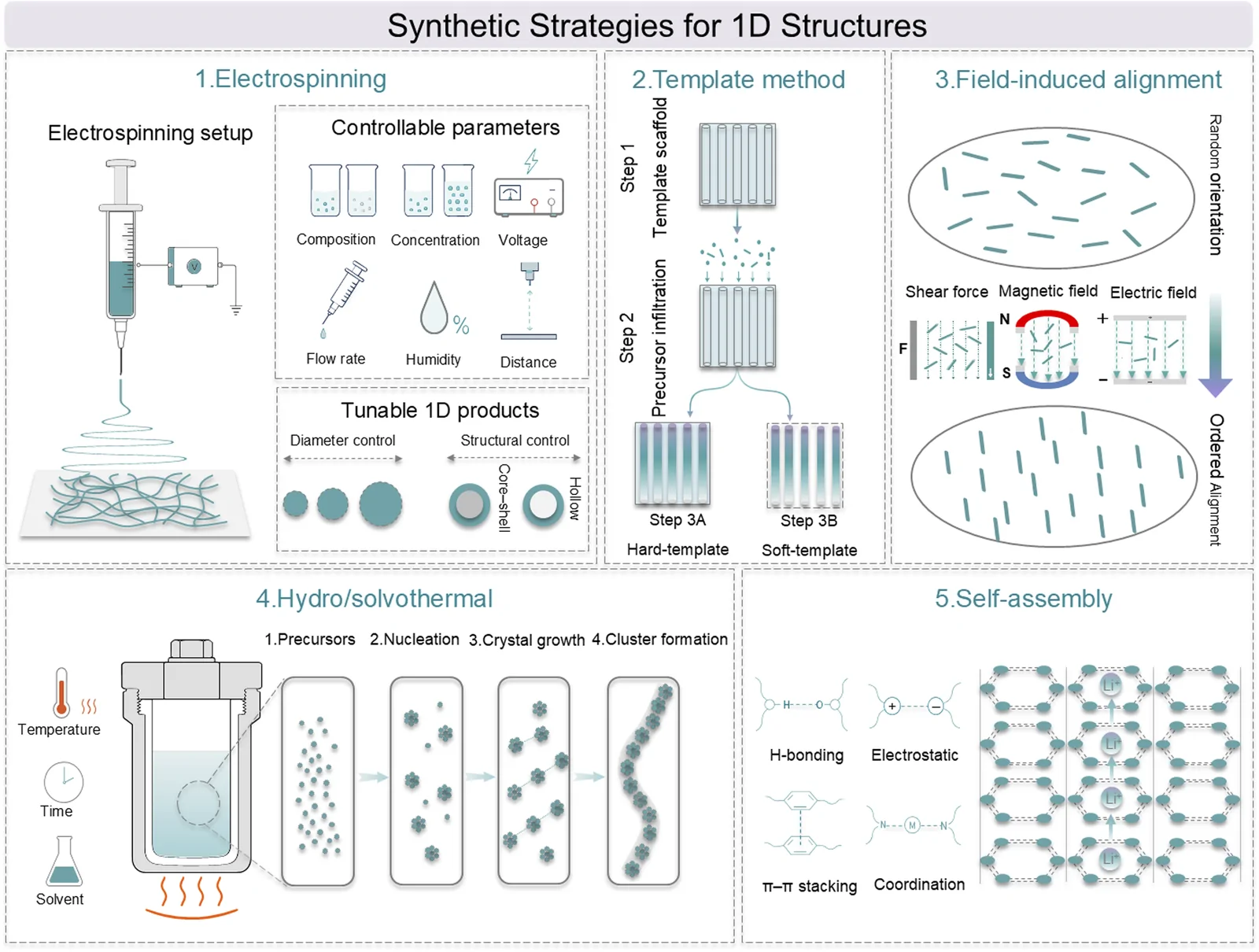
Schematic illustration of construction strategies for 1D architectures in polymer electrolytes.

The function of 1D architectures is closely related to their fabrication routes. By controlling geometry, continuity, orientation, and interfacial configuration, different methods enable distinct forms of Li^+^ transport regulation. This section summarizes representative construction strategies, including electrospinning, template-assisted methods, external-field-induced alignment, hydrothermal/solvothermal growth, and self-assembly (Fig. 4).

### 3.1 Electrospinning

**Fig. 5:**
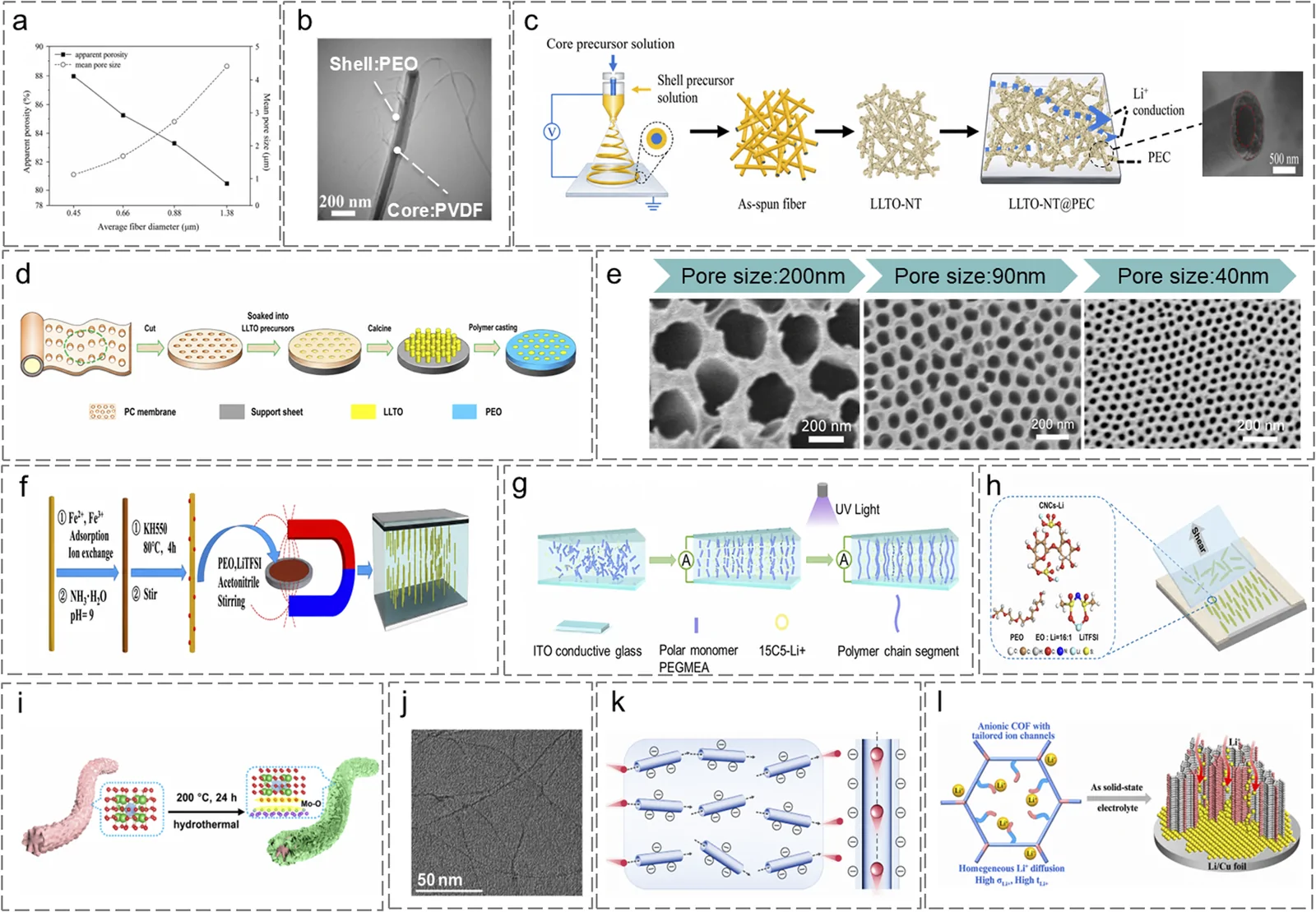
Fabrication strategies for 1D architectures in polymer electrolytes. (a) Electrospinning parameter tuning. Reproduced with permission.^63^ Copyright 2004, Elsevier. (b) Core–shell PVDF–PEO nanofibers. Reproduced with permission.^64^ Copyright 2021, Elsevier. (c) Electrospinning-assisted hollow fiber construction. Reproduced with permission.^65^ Copyright 2023, Wiley-VCH. (d) Soft-template confined growth. Reproduced with permission.^69^ Copyright 2025, Royal Society of Chemistry. (e) Hard-template pore-size control. Adapted with permission.^70^ Copyright 2018, American Chemical Society. (f) Magnetic-field-induced alignment. Reproduced with permission.^74^ Copyright 2021, Elsevier. (g) Electric-field-induced alignment. Reproduced with permission.^75^ Copyright 2024, Elsevier. (h) Shear-induced alignment. Reproduced with permission.^76^ Copyright 2025, Royal Society of Chemistry. (i) Hydrothermal heterostructure growth. Reproduced with permission.^80^ Copyright 2025, Wiley-VCH. (j) TEM image of GdOOH cluster chains. Reproduced with permission.^81^ Copyright 2023, Wiley-VCH. (k) Supramolecular self-assembly. Reproduced with permission.^86^ Copyright 2024, Wiley-VCH. (l) Schiff-base COF self-assembly. Reproduced with permission.^87^ Copyright 2024, Science China Press.

Electrospinning uses a high-voltage electrostatic field to stretch a polymer fluid into continuous 1D fibers. It is one of the representative methods for constructing 1D architectures.^61^ It features a simple setup and straightforward operation. More importantly, electrospinning allows control over nanofiber size and structure by tuning the spinning solution and processing parameters. This offers design flexibility for constructing continuous ion transport channels.^62^ J. R. Kim *et al.*^63^ first used electrospinning to prepare PVDF-HFP-based polymer electrolytes. By using PVDF solutions with concentrations of 12, 14, 16, and 18 wt%, they obtained fibrous membranes with average fiber diameters of 0.45–1.38 µm and average porosities of 80–89% (Fig. 5a). Beyond diameter and porosity control, electrospinning also enables the fabrication of structurally complex nanofibers. Gao *et al.*^64^ prepared core–shell PVDF–PEO nanofiber membranes by tuning the spinning solution and precursor flow rate. The PVDF core provided mechanical strength, whereas the PEO shell enabled Li^+^ conduction (Fig. 5b). Zhao *et al.*^65^ reported an LLTO-NT@PEC hybrid polymer electrolyte by integrating hollow LLTO nanotube frameworks with a polycarbonate-based matrix (Fig. 5c). The nanotube framework provided continuous inorganic Li^+^ pathways and mechanical reinforcement.

### 3.2 Template method

The template method directs the growth of target materials by using templates with predefined morphologies and dimensions.^66^ The template method can be divided into soft-template and hard-template approaches. After formation, the template can be retained or removed to obtain the desired structure.^67^ The key advantage of the template method is that it can predefine the size and orientation of ion transport channels at the nanoscale, thereby enabling structural regulation of 1D ion transport pathways.^68^ Li *et al.*^69^ used a porous polycarbonate (PC) membrane as a sacrificial template to confine the LLTO precursor in vertical pores. After calcination, vertically aligned LLTO nanobundle arrays were obtained and infiltrated with PEO–LiTFSI to form a composite polymer electrolyte (Fig. 5d). This structure shortened Li^+^ transport pathways and showed a room-temperature ionic conductivity of 5.6 × 10^−5^ S cm^−1^. The template method can also control the size of vertical channels. Zhang *et al.*^70^ obtained templates with pore diameters of 40, 90, and 200 nm by tuning the anodization parameters. The templates were then combined with PEO–LiTFSI (Fig. 5e). As the pore diameter decreased, the ionic conductivity of the polymer electrolyte increased markedly.

### 3.3 External-field induction

The external-field-induced method is a strategy for directing the alignment and assembly of inorganic fillers, polar molecules, or functional units under electric, magnetic, or shear fields.^71–73^ Unlike the template method, external-field induction does not require a preconstructed physical template. Instead, it directly induces directional alignment of precursor components during electrolyte film formation or curing. Han *et al.*^74^ magnetized sepiolite nanowires with Fe_3_O_4_ and then incorporated them into PEO matrix. During film formation, a magnetic field was applied to induce filler alignment (Fig. 5f). Zhao *et al.*^75^ first allowed LiTFSI to coordinate with 15-crown-5 ether (15C5) to form polarizable dipoles. A direct current electric field was then applied between indium tin oxide (ITO) electrodes to align the 15C5-Li^+^ dipoles and poly(ethylene glycol) methyl ether acrylate (PEGMEA) monomers along the field direction. The oriented structure was subsequently fixed into a three-dimensional cross-linked network by *in situ* UV polymerization. This vertical molecular orientation produced continuous Li^+^ diffusion pathways and improved cycling stability (Fig. 5g). Yu *et al.*^76^ used shear during blade coating to align lithiated cellulose nanocrystals (CNCs-Li) in a PEO/CNCs-Li/LiTFSI slurry. The directional shear flow oriented the rod-like CNCs-Li along the coating direction, and subsequent vacuum drying fixed the aligned structure (Fig. 5h). This structure formed planar Li^+^ transport pathways and improved ion transport.

### 3.4 Hydrothermal and solvothermal methods

Hydrothermal and solvothermal methods directly generate inherently 1D architectures, such as nanowires and nanorods.^77^ These methods follow a bottom-up construction route and can produce 1D architectures with well-defined crystal orientations and tunable surface chemistry.^78,79^ Shan *et al.*^80^ grew MoSe_2_*in situ* on BaTiO_3_ fibers by a hydrothermal method to form heterointerfaces. The n-type BaTiO_3_ and p-type MoSe_2_ generated a built-in electric field at the interface, promoting lithium salt dissociation (Fig. 5i). Cheng *et al.*^81^ used oleylamine and oleic acid to restrict the lateral growth of GdOOH nuclei and stabilize their surfaces, leading to the formation of ultrafine 1D GdOOH cluster chains (Fig. 5j). The sub-1 nm dispersion improved compatibility with the PEO–LiTFSI matrix and reduced Li^+^-inactive regions caused by filler aggregation.

### 3.5 Self-assembly method

Self-assembly relies on hydrogen bonding, electrostatic interactions, π–π stacking, coordination interactions, or microphase separation of block copolymers to form linear nanostructures within the polymer matrix.^82,83^ Self-assembly relies on intrinsic interactions among building units to form ordered structures spontaneously, which helps improve compatibility and reduce structural defects.^84,85^ Li *et al.*^86^ used noncovalent interactions to drive the spontaneous assembly of cucurbit^7^ uril (CB^7^) molecules into ordered pore channels. These channels constructed 1D Li^+^ migration pathways at the molecular scale (Fig. 5k). Meanwhile, hydrogen bonding immobilized PF_6_^−^ anions, leading to a high Li^+^ transference number and stable Li deposition. Zhuang *et al.*^87^ introduced a sulfonate-grafted covalent organic framework (COF), denoted as COF-S, into an ethoxylated trimethylolpropane triacrylate (ETPTA)/lithium bis(fluorosulfonyl)imide (LiFSI) gel electrolyte. COF-S was synthesized through a Schiff base reaction, in which aldehyde groups condensed with amino groups to form imine bonds (C=N) (Fig. 5l). These C=N bonds connected the monomers into an ordered framework with directional channels. The SO_3_^−^ groups in the channels provided Li^+^ adsorption and hopping sites, thereby constructing ordered Li^+^ transport channels at the molecular scale.

These strategies differ not only in mechanism but in the trade-off each strikes between structural precision and practical throughput. Electrospinning affords broad morphological control, extending readily to core–shell and hollow variants, and integrates naturally with continuous processing. Its main limitation is that fiber diameter and porosity remain governed largely by empirical parameter tuning rather than direct structural design. Template-directed synthesis achieves the most precise command over channel geometry, a precision well suited to mechanistic studies. However, the required template-removal step adds complexity and constrains throughput. External-field induction occupies a distinct niche. It aligns pre-formed fillers without altering their intrinsic chemistry, making it well suited when directional transport is the primary design objective. The resulting alignment, though, can be difficult to preserve under the shear and thermal stresses of battery assembly. Hydrothermal and solvothermal growth excel at tailoring the crystal structure and surface chemistry of intrinsically conductive ceramic fillers, at the expense of batch-wise, energy-intensive processing. Self-assembly is unique in producing molecular-scale ordered channels without any inorganic filler, but it depends on stringent molecular design and remains the least scalable of the five routes. The choice of construction method should be guided by the targeted structure and function. Representative fabrication strategies for 1D architectures in polymer electrolytes are summarized in Table 1.

**Table 1:** Fabrication strategies for 1D architectures in polymer electrolytes^a^

Strategy	Scalability	Polymer matrix	Structure	Ref.
Electrospinning	High	PVDF-HFP	Common nanofibers	63
Electrospinning	High	PVDF/PEO	Core–shell nanofibers	64
Electrospinning	High	PEC	Hollow nanofibers	65
Soft template	Low	PEO	Aligned nanotube	69
Hard template	Medium	PEO	Aligned channels	70
Magnetic field	Low	PEO	Aligned nanotube	74
Electric field	Low	PEGMEA	Aligned molecular	75
Shear field	Medium	PEO	Aligned nanocrystals	76
Hydrothermal	Medium	PVDF	Heterofibers	80
Solvothermal	Medium	PEO	Ultrafine nanowires	81
Self-assembly	Low	ETPTA	Molecularly aligned channels	87

a Scalability is rated qualitatively by throughput and compatibility with continuous roll-to-roll battery manufacturing: high, continuous and readily scalable; medium, semi-continuous or batch-wise but adaptable; low, batch-limited or requiring stringent processing conditions.

## 4 How 1D architectures regulate polymer electrolytes

1D architectures can interfere with polymer crystallization in polymer electrolytes. Xia *et al.*^88^ observed sharp crystalline peaks at 19.3° and 23.4° for pristine PEO, whereas the PI nanofiber composite showed only broad amorphous features. This suggests that 1D fibers disrupted PEO crystallization and inhibited the growth of continuous crystalline domains. Han *et al.*^89^ incorporated Al-doped LLZO nanofibers into PVDF. The decrease in melting temperature from 143.0 to 139.6 °C suggests that the nanofibers weakened polymer chain packing. However, crystallinity reduction is a general effect of fillers rather than a feature unique to 1D architectures. Therefore, the following discussion focuses on how 1D architectures reconstruct transport pathways and enhance the role of interfacial functions. The unique role of 1D architectures arises from their spatial continuity and directionality. These features help regulate Li^+^ transport paths, extend interfacial effects, and improve stress transfer. Accordingly, the following sections focus on these structure-dependent advantages of 1D architectures.

### 4.1 Structure-directed Li^+^ transport

**Fig. 6:**
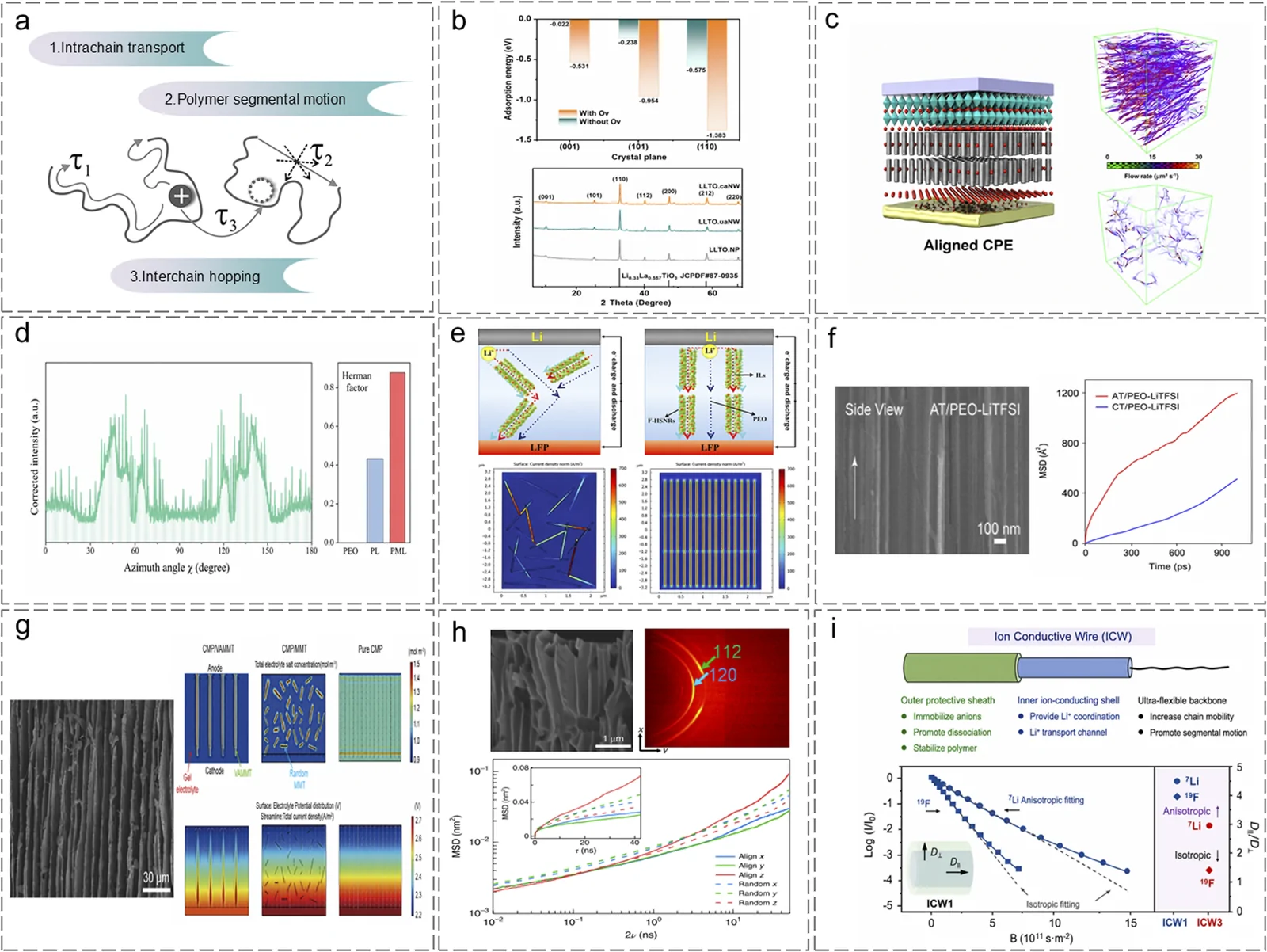
Directional Li^+^ transport enabled by oriented 1D architectures in polymer electrolytes. (a) Conventional segmental Li^+^ transport. Adapted with permission.^90^ Copyright 2007, American Physical Society. (b) DFT-calculated adsorption behavior of PVP on LLTO and XRD for LLTO-caNWs in polymer electrolytes. Adapted with permission.^93^ Copyright 2024, American Chemical Society. (c) Schematic illustration and simulated ion-flux distribution of HNTs–SO_3_Li in polymer electrolytes. Adapted with permission.^94^ Copyright 2025, Wiley-VCH. (d) Azimuthal WAXS profile and Hermans orientation factor of shear-aligned MCM-22 lamellae in polymer electrolytes. Adapted with permission.^95^ Copyright 2026, Elsevier. (e) Schematic illustrations and COMSOL simulations of randomly dispersed and vertically aligned F-ILs@HSNRs in polymer electrolytes. Adapted with permission.^96^ Copyright 2025, Wiley-VCH. (f) Scanning electron microscopy (SEM) image and molecular dynamics simulation of Li^+^ transport in polymer electrolytes with AT arrays. Adapted with permission.^97^ Copyright 2025, Wiley-VCH. (g) SEM image and COMSOL simulation of CMP/VAMMT, CMP/MMT, CMP. Adapted with permission.^98^ Copyright 2022, Springer Nature. (h) SEM image, 2D WAXS pattern, and molecular dynamics simulation of PI channels guided PEO chain orientation. Adapted with permission.^99^ Copyright 2019, Springer Nature. (i) Structural design and PFG-NMR analysis of ICWs. Reproduced with permission.^100^ Copyright 2026, American Chemical Society.

In typical PEO-LiTFSI electrolytes, Li^+^ transport is closely coupled with the segmental relaxation of PEO chains and proceeds through repeated decoordination and recoordination between ether oxygen sites.^90^ Because the orientation of polymer chains is disordered and the transport sites are randomly distributed in space, Li^+^ migration pathways usually have high tortuosity (Fig. 6a).^91,92^

This suggests that improving Li^+^ transport efficiency in polymer electrolytes requires reducing migration disorder. Based on this concept, constructing 1D architectures within polymer matrices is considered an effective way to reduce the tortuosity of Li^+^ transport pathways. In this section, 1D architectures are discussed as structural platforms for directing Li^+^ migration rather than merely as filler morphologies. Kou *et al.*^93^ first demonstrated the importance of matching the 1D architectures with the intrinsic fast Li^+^ migration direction of ceramic fillers. Density functional theory (DFT) calculations showed that polyvinylpyrrolidone (PVP) had the strongest adsorption on the LLTO (110) plane, which enabled a 1D PVP sheath to induce the preferential growth of *c*-axis-aligned lithium lanthanum titanate nanowires (LLTO-caNWs) during sintering. X-ray diffraction (XRD) confirmed enhanced *c*-axis orientation, aligning the nanowire direction with the fastest Li^+^ migration pathway in LLTO. After incorporation into PEO–LiTFSI, the electrolyte delivered ionic conductivity of 1.29 × 10^−4^ S cm^−1^ at 25 °C, much higher than that of the electrolyte containing LLTO nanoparticles (Fig. 6b). This direction-matching strategy can be further combined with surface functionalization and external-field alignment. Peng *et al.*^94^ modified halloysite nanotubes with –SO_3_Li groups (HNTs–SO_3_Li) and used electric field to induce their axial alignment in polyurethane acrylate (PUA)/PEGDA matrix. The negatively charged –SO_3_Li sites adsorbed Li^+^ and promoted directional ion migration. Simulated ion-flux distributions showed that the ion-flux nonuniformity factor decreased from 0.41 in the pristine composite polymer electrolyte (CPE) to 0.13 in the aligned CPE, confirming more ordered Li^+^ migration and a more uniform ion flux (Fig. 6c).

Beyond individual nanowire design, macroscopic alignment also plays a decisive role in reducing Li^+^ transport tortuosity. Yuan *et al.*^95^ achieved oriented Li^+^ transport by shear-aligning exfoliated MCM-22 zeolite lamellae (MCM-22 lamellae) in PEO matrix. The azimuthal intensity profile extracted from the two-dimensional wide-angle X-ray scattering (WAXS) pattern showed two sharp and symmetric peaks at approximately 45° and 135°, indicating preferred lamellar orientation rather than random dispersion. This result was further supported by a high Hermans orientation factor of 0.91. The oriented lamellar arrangement reduced the tortuosity of Li^+^ migration pathways, as reflected by the decreased activation energy from 0.9 to 0.77 eV (Fig. 6d). Similarly, Gao *et al.*^96^ constructed an oriented 1D ion-transport architecture by magnetically aligning hollow silicon nanorods containing ionic liquids (F-ILs@HSNRs) in PEO matrix. COMSOL simulations revealed that the perpendicular arrangement provided shorter Li^+^ migration pathways than random or parallel configurations, giving a high ionic conductivity of 2.14 × 10^−4^ S cm^−1^ (Fig. 6e).

Oriented array structures further highlight the importance of through-thickness ion migration. Hou *et al.*^97^ developed amorphous titanium dioxide nanotube arrays (AT arrays) in PEO–LiTFSI, where the vertically arranged nanotubes promoted Li^+^ migration across the membrane. Molecular dynamics simulations showed that the Li^+^ diffusion coefficient in the AT-based electrolyte reached 0.16 Å^2^ ps^−1^ (Fig. 6f). Li *et al.*^98^ constructed vertically aligned montmorillonite arrays (VAMMT) in CMP matrix. COMSOL simulations showed that Li^+^ was enriched and migrated along the vertical VAMMT arrays, whereas randomly dispersed MMT only induced local ion enrichment and failed to regulate Li^+^ transport effectively (Fig. 6g). This pathway-regulation principle is not limited to inorganic 1D fillers. Wan *et al.*^99^ used polyimide channels (PI channels) to orient PEO chains. In the two-dimensional WAXS pattern, the diffraction intensity of the PI/PEO/LiTFSI film was concentrated along the *y* direction, confirming PEO segment alignment along the PI channels. Molecular dynamics simulations gave Li^+^ diffusion coefficient of 1.3 × 10^−8^ cm^2^ s^−1^ along the channel direction, higher than 5.7 × 10^−9^ cm^2^ s^−1^ in the disordered system (Fig. 6h). More recently, Han *et al.*^100^ reported self-assembled ion-conductive wires (ICWs) as polymeric 1D architectures for structure-directed Li^+^ transport. Pulsed-field-gradient nuclear magnetic resonance (PFG-NMR) revealed strong anisotropic ^7^Li diffusion in ICW1, confirming preferential Li^+^ migration along the wire axis. ICW1 achieved an ionic conductivity of 1.8 × 10^−4^ S cm^−1^ and activation energy of 0.11 eV (Fig. 6i). Representative 1D architectures that reduce Li^+^ transport tortuosity are summarized in Table 2.

**Table 2:** Representative 1D architectures for low-tortuosity Li^+^ transport^a^

Structure	Polymer matrix	State	Key evidence	*σ*_RT_ [S cm^−1^]	Ref.
LLTO-caNWs	PEO	Dry SPE	DFT, XRD	1.29 × 10^−4^	93
HNTs–SO_3_Li	PUA/PEGDA	Dry SPE	Ion-flux simulation	4.89 × 10^−4^	94
MCM-22 lamellae	PEO	Dry SPE	WAXS	5.4 × 10^−4^	95
F-ILs@HSNRs	PEO	Gel	COMSOL simulation	2.14 × 10^−4^	96
AT arrays	PEO	Gel	SEM, MSD simulation	1.34 × 10^−4^	97
VAMMT	PEO	Gel	SEM, COMSOL simulation	1.08 × 10^−3^	98
PI channels	PEO	Dry SPE	SEM, WAXS, MSD simulation	2.3 × 10^−4^	99
ICWs	PEG/PDMS	Dry SPE	PFG-NMR	1.8 × 10^−4^	100

a State: “Dry SPE” denotes an all-solid electrolyte free of a mobile liquid phase; “Gel” denotes systems containing a liquid electrolyte, ionic liquid, or otherwise gel-type phase.

### 4.2 Long-range interfacial regulation

Interfaces should not be viewed only as phase boundaries. They can also serve as active functional regions that regulate polymer chain conformation, promote ion dissociation, and participate in Li^+^ transport.^101^ For example, Liu *et al.*^102^ used molecular sieves rich in interfacial Lewis acidic sites to regulate Li^+^ coordination and lithium salt dissociation in PEO–LiTFSI. Liu *et al.*^103^ revealed interfacial Li^+^ exchange between PEO and Li_6_PS_5_Cl, and Wu *et al.*^104^ demonstrated rapid Li^+^ migration along ceramic/polymer interfaces. These studies show that interfaces can actively participate in ion transport, but such effects are often limited to local regions around isolated fillers.

**Fig. 7:**
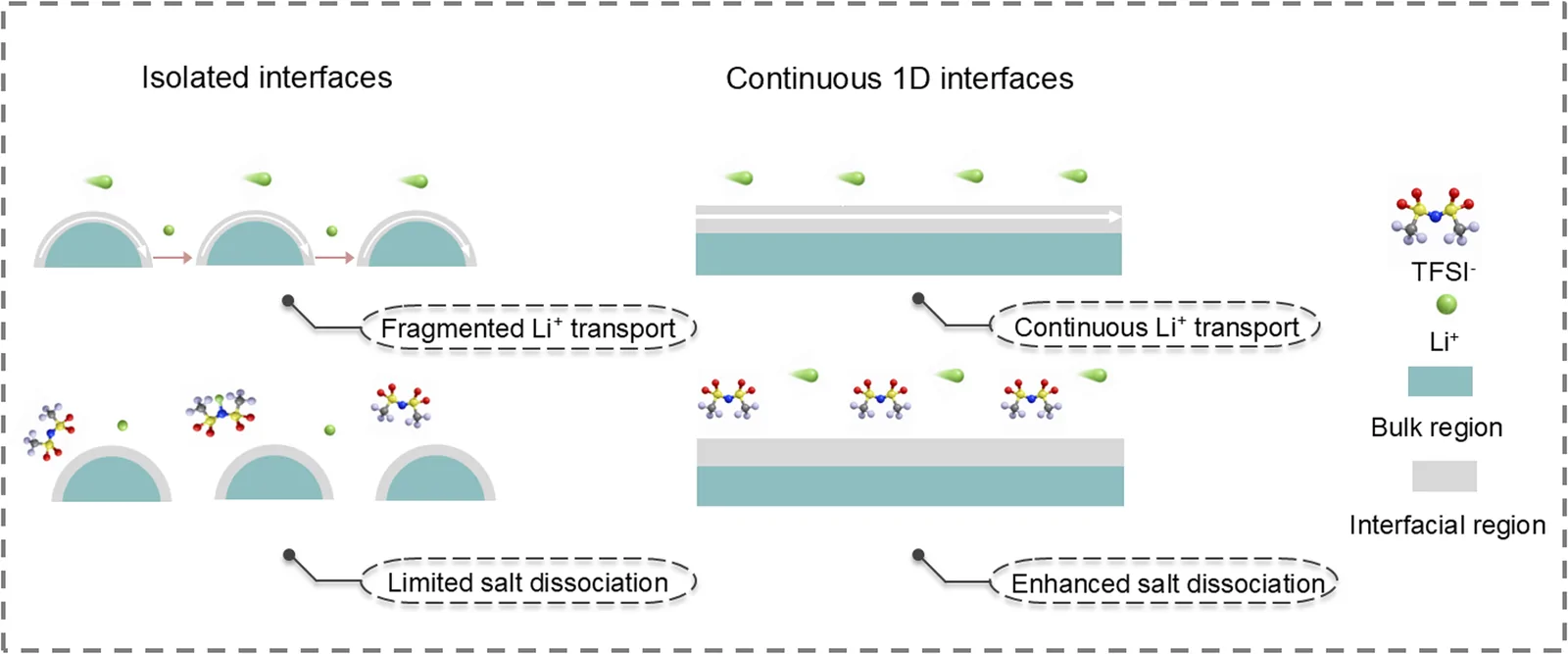
Continuous 1D interfaces for coupled Li^+^ transport and salt dissociation.

1D architectures provide a way to extend these local interfacial effects into continuous pathways. By improving interfacial continuity, they allow lithium salt dissociation, anion immobilization, and Li^+^ transport to occur along the axial direction rather than only around isolated filler surfaces (Fig. 7).

#### 4.2.1 Continuous interfacial Li^+^ transport

1D architectures can promote Li^+^ transport through extended interfaces. These interfaces provide additional Li^+^ coordination and migration regions, converting local filler effects into effective interfacial transport pathways.

**Fig. 8:**
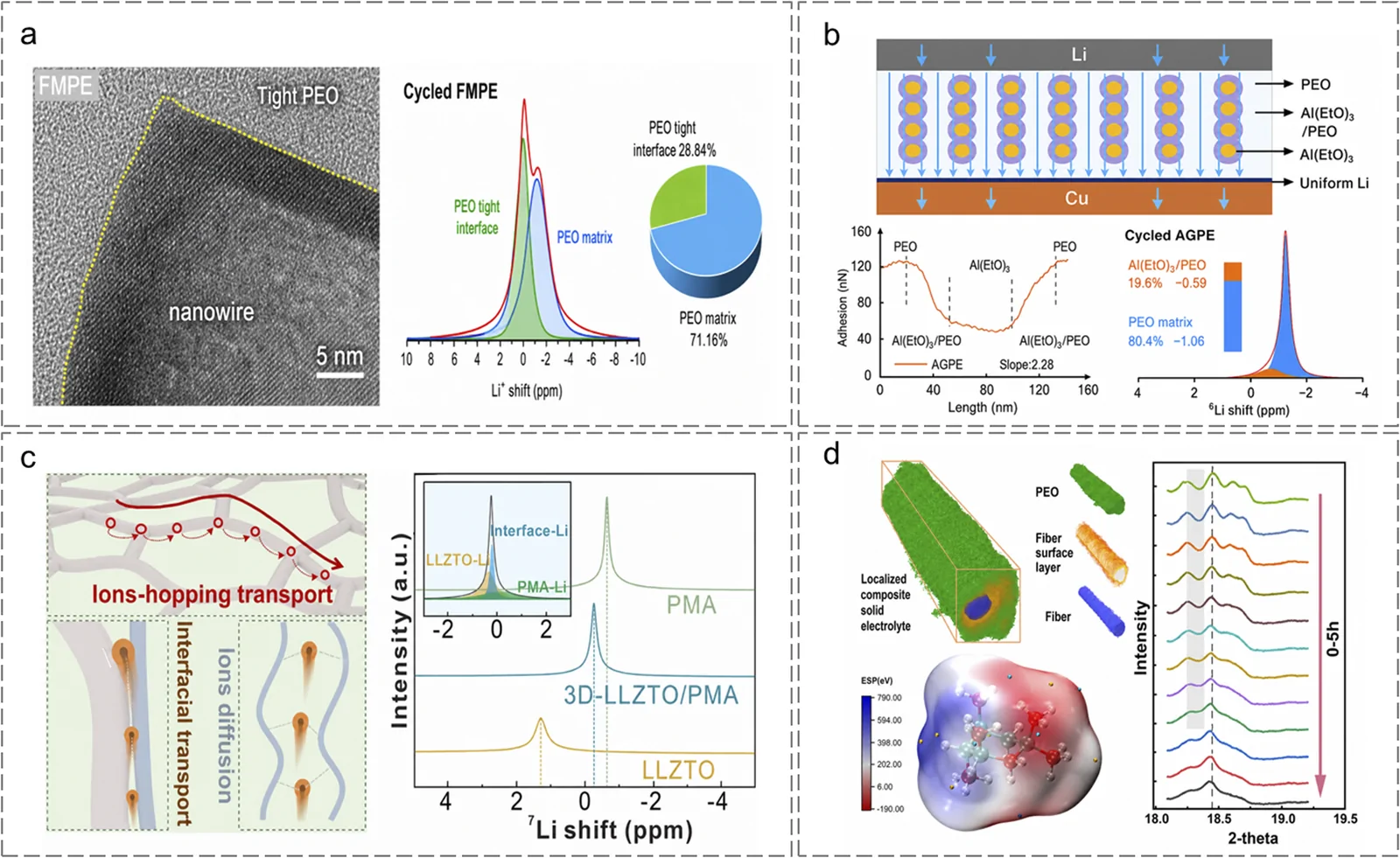
Continuous interfacial Li^+^ transport enabled by 1D architectures in polymer electrolytes. (a) TEM image and ^6^Li NMR analysis of Li^+^ transport in Mn-doped MgAl_2_O_4_ NW-based electrolytes. Adapted with permission.^106^ Copyright 2022, Wiley-VCH. (b) Schematic illustration, AFM adhesion mapping, and ^6^Li NMR analysis of Li^+^ transport in Al(EtO)_3_ NW-based electrolytes. Adapted with permission.^107^ Copyright 2024, Elsevier. (c) Schematic illustration and ^7^Li NMR analysis of Li^+^ transport in Ta-doped LLZTO NW-based electrolyte. Adapted with permission.^108^ Copyright 2026, Elsevier. (d) Electrostatic potential mapping and *operando* WAXS analysis of Li^+^ transport in (CNFs)@PEO. Adapted with permission.^109^ Copyright 2026, Wiley-VCH.

Compared with zero-dimensional nanoparticles, high aspect ratio 1D fillers more readily form continuous ion transport pathways. A direct comparison was reported by Yang *et al.*,^105^ who incorporated LLZO nanowires and LLZO nanoparticles into PAN-based electrolytes. The LLZO nanowire/PAN electrolyte delivered a room-temperature ionic conductivity of 1.31 × 10^−4^ S cm^−1^, which was about 11.6 times higher than that of the LLZO nanoparticle counterpart, 1.13 × 10^−5^ S cm^−1^, even though the nanoparticles had a smaller particle size of approximately 25 nm. This comparison indicates that the transport enhancement is not determined only by particle size, but is strongly related to the spatial continuity of the filler/polymer interface. This view is supported by direct evidence of Li^+^-rich interfacial regions along 1D fillers. Ye *et al.*^106^ showed that Mn-doped MgAl_2_O_4_ nanowires induced continuous interfacial layers on their surfaces. ^6^Li nuclear magnetic resonance (NMR) revealed that 28.84% of Li^+^ resided in the intimate 1D interfacial regions, supporting interfacial Li^+^ conduction. As a result, FMPE achieved a high ionic conductivity of 5.66 × 10^−4^ S cm^−1^ at 25 °C (Fig. 8a). This interfacial transport mechanism was further verified by Ou *et al.*,^107^ who incorporated Al(EtO)_3_ nanowires into poly(ethylene glycol) diglycidyl ether (PEGDE). Atomic force microscopy (AFM) adhesion mapping revealed intimate nanowire/polymer interfaces, while ^6^Li NMR confirmed long-distance Li^+^ transport within the interfacial regions (Fig. 8b). Together, these results demonstrate that chemically coupled 1D interfaces can provide additional Li^+^ migration pathways beyond the bulk polymer phase. Similar interfacial transport behavior can also be extended to continuous ceramic frameworks coupled with polymer matrices. Xu *et al.*^108^ constructed a composite electrolyte by integrating Ta-doped lithium lanthanum zirconium oxide (LLZTO) framework with poly(methyl acrylate) (PMA) polymer matrix. As shown in Fig. 8c, the ^7^Li NMR chemical shift of LLZTO/PMA lies between those of PMA and LLZTO, confirming that the ceramic/polymer interface actively participates in Li^+^ migration. This interfacial transport contribution is supported by the reduced activation energy of 0.360 eV and the ionic conductivity of 1.08 × 10^−4^ S cm^−1^.In addition to inorganic interfaces, bio-derived 1D interfaces can regulate Li^+^ migration by reshaping the local polymer phase. Cui *et al.*^109^ constructed Apocynum venetum-derived cellulose nanofibers (CNFs) in PEO-based polymer electrolytes. The electrostatic potential (ESP) map revealed abundant Li^+^ binding sites on the CNF surface, indicating that the interface provides favorable coordination environments for Li^+^ migration. In addition, the disappearance of the WAXS diffraction peak near 2*θ* ≈ 18.15° confirmed that CNFs promote PEO amorphization near the fiber surface. The resulting amorphous interfacial regions induce Li^+^ conduction pathways and reduce the activation energy to 0.379 eV (Fig. 8d).

Overall, 1D architectures enhance ion transport by transforming isolated contacts between fillers and polymers into continuous Li^+^ migration pathways, thereby reducing pathway discontinuity and strengthening interfacial Li^+^ conduction. Representative systems demonstrating continuous interfacial Li^+^ transport are compared in Table 3.

**Table 3:** Representative 1D architectures for continuous interfacial Li^+^ transport^a^

Structure	Polymer matrix	State	Key evidence	*σ*_RT_ [S cm^−1^]	*E*_a_ [eV]	Ref.
LLZO NWs	PAN	Dry SPE	^6^Li NMR	1.31 × 10^−4^	0.12	105
Mn-doped MgAl_2_O_4_ NWs	PEO	Dry SPE	TEM, ^6^Li NMR	5.66 × 10^−4^	0.235	106
Al(EtO)_3_ NWs	PEO	Dry SPE	AFM, ^6^Li NMR	6.8 × 10^−4^	0.229	107
Ta-doped LLZTO NWs	PMA	Dry SPE	^7^Li NMR	1.08 × 10^−4^	0.36	108
CNFs	PEO	Dry SPE	ESP map, WAXS	6.65 × 10^−5^	0.379	109

a State: see footnote to Table 2.

#### 4.2.2 Amplified interfacial chemical regulation

Extended interfacial regions can amplify the functional role of 1D architectures beyond simple pathway construction. By providing spatially continuous surface sites, these architectures promote lithium salt dissociation, regulate anion coordination, and improve the selectivity of Li^+^ migration.

**Fig. 9:**
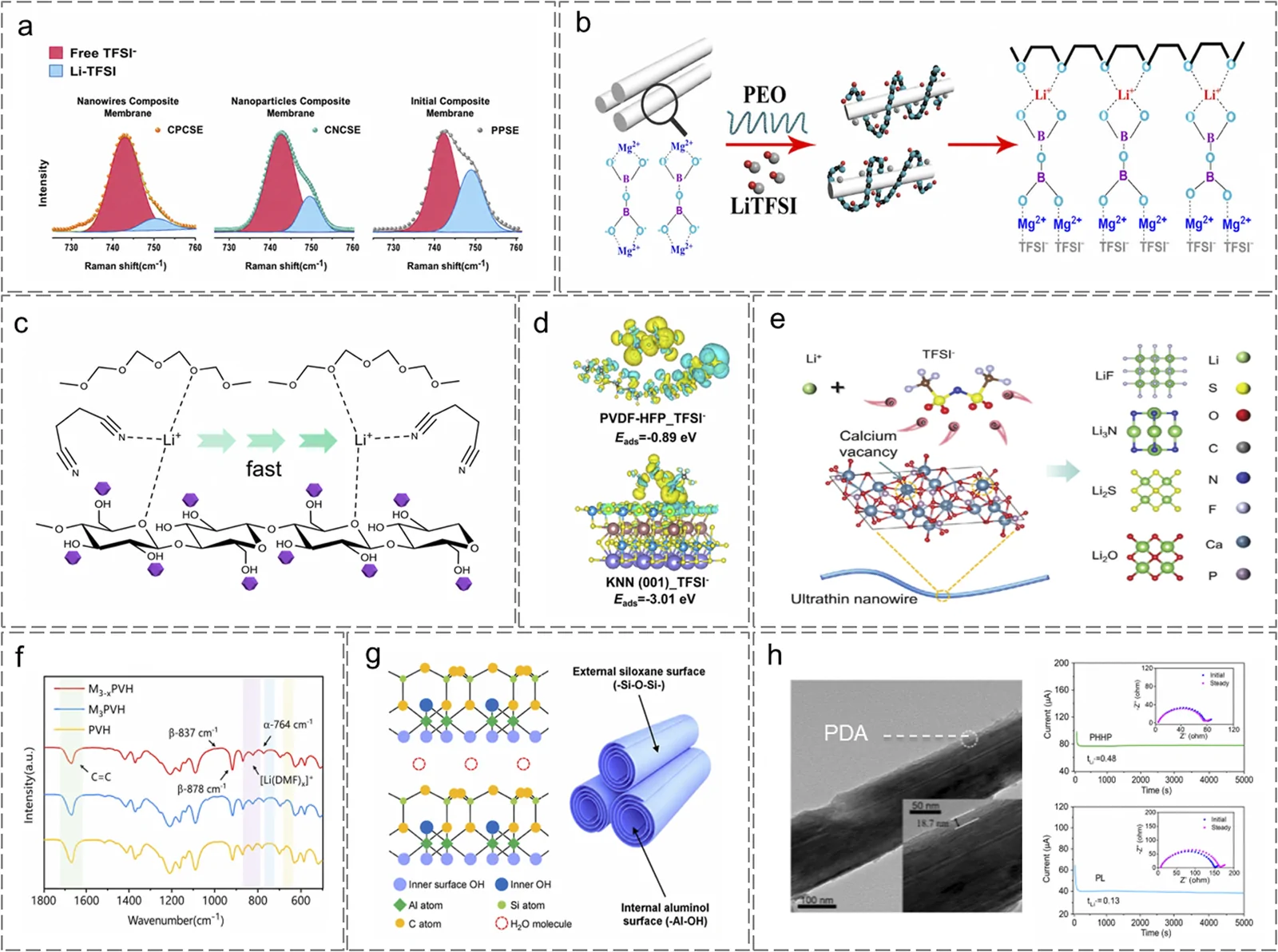
Amplified interfacial chemical regulation by 1D architectures in polymer electrolytes. (a) Raman analysis of GdOOH NWs in electrolytes. Adapted with permission.^81^ Copyright 2023, Wiley-VCH. (b) Schematic illustration of Mg_2_B_2_O_5_ NWs interacting with TFSI^−^. Reproduced with permission.^110^ Copyright 2018, American Chemical Society. (c) Schematic illustration of Li^+^ coordination and interfacial hopping along ZIF-67@CF. Reproduced with permission.^111^ Copyright 2024, American Chemical Society. (d) Adsorption energy of TFSI^−^ on PVDF-HFP and KNN(001). Reproduced with permission.^112^ Copyright 2026, Wiley-VCH. (e) Interfacial catalytic decomposition of TFSI^−^ on CHANWs with Ca vacancies. Adapted with permission.^113^ Copyright 2024, Wiley-VCH. (f) FTIR spectra of PVH, M_3_PVH, and M_3−*x*_PVH electrolytes. Reproduced with permission.^114^ Copyright 2025, Wiley-VCH. (g) Schematic structure of HNTs with asymmetric inner and outer surfaces for Li^+^-selective transport. Reproduced with permission.^115^ Copyright 2026, American Chemical Society. (h) TEM image of PDA@HNTs, chronoamperometry and impedance analysis of PHHP and PL electrolytes. Adapted with permission.^116^ Copyright 2025, Elsevier.

A representative example is the use of 1D Lewis acidic chains to enhance salt dissociation. Cheng *et al.*^81^ incorporated monodisperse GdOOH cluster chains into PEO–LiTFSI. The cluster chains exposed more Lewis acidic sites than isolated GdOOH nanoparticles and showed stronger TFSI^−^ adsorption. Raman results indicated the highest free TFSI^−^ fraction in the cluster chain composite electrolyte (Fig. 9a). Sheng *et al.*^110^ incorporated Mg_2_B_2_O_5_ nanowires into PEO–LiTFSI polymer electrolyte. The nanowires constructed continuous interfacial regions that interacted with TFSI^−^, promoted lithium salt dissociation, and increased the Li^+^ transference number from 0.19 to 0.44 (Fig. 9b).

Such Lewis acid–base regulation can also be extended to polarized and functionalized 1D interfaces that jointly modulate anion anchoring and Li^+^ coordination. For example, Song *et al.*^111^ incorporated ZIF-67-functionalized cellulose fiber skeleton (ZIF-67@CF) in PEO-based electrolytes. Unlike isolated filler contacts, the continuous fiber interfaces distribute polar coordination sites along the 1D framework, weakening Li^+^–PEO interactions and enabling interfacial Li^+^ hopping. This promotes faster Li^+^ migration along the functionalized fiber interface (Fig. 9d). Similarly, Jia *et al.*^112^ introduced potassium sodium niobate-containing electrospun fibrous framework (KNN fibrous framework) to regulate interfacial ion chemistry in PVDF-HFP-based electrolytes. As shown in Fig. 9c, TFSI^−^ shows much stronger charge redistribution on the KNN(001) surface than on PVDF-HFP. This surface promotes LiTFSI dissociation and facilitates Li^+^ transport.

Defect-rich 1D interfaces can further regulate anion decomposition and interphase chemistry. Yang *et al.*^113^ further developed an interfacial catalytic strategy using ultrafine CHANWs. The 1.5–2.0 nm nanowires formed abundant inorganic–organic interfaces in the polymer. Ca-vacancy-related unpaired electrons promoted TFSI^−^ decomposition and induced inorganic-rich SEI, enabling long-distance regulation of lithium salt decomposition (Fig. 9e). Xue *et al.*^114^ found that oxygen-vacancy-rich MoO_3−*x*_ nanowires could regulate the local solvation environment in a PVDF-HFP-based electrolyte through continuous surface oxygen vacancy sites. FTIR spectra showed that the C=O absorption peak of DMF in the nanowire composite electrolyte (M_3−*x*_PVH) shifted from 1655 to 1660 cm^−1^. Meanwhile, the intensity of the [Li(DMF)_*x*_]^+^ characteristic peak decreased. These results indicate that the nanowires weakened Li^+^–DMF coordination and promoted lithium salt dissociation (Fig. 9f). Hollow tubular 1D architectures provide another platform for interfacial ion selectivity. Na *et al.*^115^ incorporated HNTs into polyurethane matrix. The asymmetric charge distribution on the inner and outer surfaces of HNTs promoted selective Li^+^ transport, giving a Li^+^ transference number of 0.767 (Fig. 9g). In a related design, Chen *et al.*^116^ used PDA-modified halloysite nanotubes. The continuous amino sites on the nanotube surface immobilized TFSI^−^ through hydrogen bonding. As a result, the Li^+^ transference number of PHHP increased to 0.48, much higher than that of PL (0.13) (Fig. 9h). Together, these studies show that the contribution of 1D architectures is not limited to reducing transport tortuosity. More importantly, their extended interfaces can transform localized Lewis acid–base interactions, defect chemistry, and surface functional groups into long-range regulation of lithium salt dissociation, anion immobilization, and Li^+^-selective transport. Representative 1D architectures for interfacial chemical regulation are summarized in Table 4.

**Table 4:** Interfacial chemical regulation by 1D architectures in polymer electrolytes^a^

Structure	Polymer matrix	State	Key evidence	*σ*_RT_ [S cm^−1^]	*t*_Li^+^_	Ref.
GdOOH NWs	PEO	Dry SPE	Raman	5.2 × 10^−4^	0.62	81
Mg_2_B_2_O_5_ NWs	PEO	Dry SPE	FTIR	1.53 × 10^−4^	0.44	110
ZIF-67@CF	PEO	Dry SPE	DFT	1.17 × 10^−4^	0.40	111
KNN fibers	PVDF-HFP	Dry SPE	DFT, XPS	6.7 × 10^−4^	0.68	112
CHANWs	PEO	Dry SPE	DFT, AIMD	6.33 × 10^−4^	N/A	113
MoO_3−*x*_ NWs	PVDF-HFP	Gel	FTIR	7.58 × 10^−4^	0.78	114
HNTs	TPU	Gel	XPS	1.04 × 10^−3^	0.767	115
PDA@HNTs	PVDF-HFP	Dry SPE	FTIR	2.4 × 10^−4^	0.48	116

a State: see footnote to Table 2.

### 4.3 Long-range stress transfer

Polymer electrolytes generally show good flexibility and interfacial compatibility, but their low modulus and limited puncture resistance make them vulnerable to dendrite penetration and local stress concentration during long-term cycling. Compared with 0D particulate fillers, 1D materials can reinforce polymer electrolytes more efficiently because their high aspect ratio and spatial continuity enable the formation of load-bearing networks.^117–119^ Unlike discrete particles that mainly cause local stiffening around fillers, continuous fibrous networks provide long-range stress transfer pathways throughout the polymer matrix.^120^ They can also dissipate fracture energy through crack bridging, fiber pull-out, and interfacial friction, thereby delaying crack propagation and structural failure.^121,122^

**Fig. 10:**
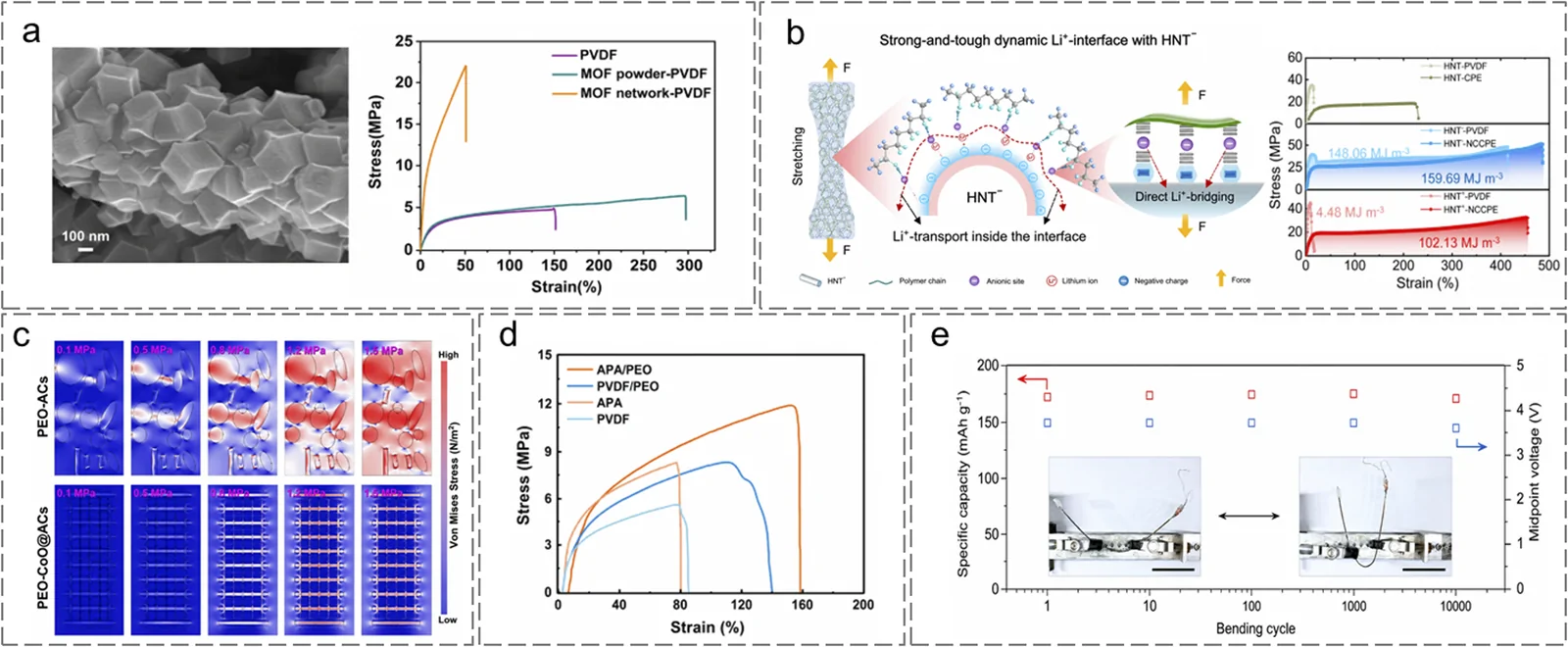
Stress transfer and mechanical reinforcement enabled by 1D frameworks in polymer electrolytes. (a) ZIF-8@PI fiber network and tensile reinforcement of PVDF electrolytes. Adapted with permission.^56^ Copyright 2022, Wiley-VCH. (b) Li^+^-mediated interfacial bridging in HNT-based composite electrolytes. Adapted with permission.^123^ Copyright 2026, Springer Nature. (c) Stress dissipation in CoO@ACs heterostructured nanotube networks. Adapted with permission.^124^ Copyright 2026, Elsevier. (d) Tensile reinforcement by aramid nanofiber-based load transfer networks. Reproduced with permission.^126^ Copyright 2026, Elsevier. (e) Bending stability of TEGDMA-based fiber-shaped lithium-ion batteries. Reproduced with permission.^127^ Copyright 2025, Wiley-VCH.

A direct comparison between discrete particles and continuous 1D networks was reported by Du *et al.*^56^ They induced ZIF-8 nanocrystals to self-assemble along polyimide fibers, forming a continuous MOF network. Compared with randomly dispersed MOF powders, the continuous coverage of MOF nanocrystals on 1D PI fibers enabled more effective stress transfer through the fiber network. As a result, the tensile strength increased from 4.98 MPa for pure PVDF and 6.41 MPa for the MOF powder composite to 22.02 MPa for MOF network-PVDF. This demonstrates that continuous 1D networks provide stronger mechanical support than discrete particles and improve dendrite resistance in composite polymer electrolytes (Fig. 10a).

In addition to network continuity, interfacial coupling between 1D fillers and polymer chains is also critical for mechanical reinforcement. Lv *et al.*^123^ used negatively charged HNTs in a PVDF-based electrolyte. The HNTs adsorbed Li^+^, which then coordinated with fluorine atoms in PVDF chains to form Li^+^-mediated bridges between the nanotubes and the polymer matrix. This interfacial bridging improved stress transfer and increased the toughness of HNTs NCCPE to 159.69 MJ m^−3^ (Fig. 10b). Mu *et al.*^124^ introduced CoO@ACs heterostructured nanotubes into a PEO electrolyte. The continuous ACs framework increased the tensile strength to 1.712 MPa, while the stress distribution simulation showed that CoO@ACs enabled more homogeneous stress dissipation under external loading. This result suggests that 1D networks can improve the mechanical robustness of polymer electrolytes by suppressing local stress concentration (Fig. 10c).

Polymeric 1D fibers can further reinforce electrolytes by forming mechanically robust frameworks. Tung *et al.*^125^ used Kevlar-derived aramid nanofibers to reinforce PEO. The interconnected 1D fiber network improved membrane stiffness and helped redistribute local stress, giving a tensile strength of 170 ± 5 MPa, a Young's modulus of 5.0 ± 0.05 GPa, and a shear modulus of 1.8 ± 0.06 GPa. Similarly, Xu *et al.*^126^ reinforced a PVDF and PEO electrolyte with aramid nanofibers. The high aspect ratio fibers formed a continuous load-bearing network through π–π stacking interactions, increasing the tensile strength from 7.79 MPa for PVDF/PEO to 11.46 MPa for APA/PEO (Fig. 10d). At the device level, the mechanical benefits of 1D architectures can be further reflected in fiber-shaped batteries. Cheng *et al.*^127^ reported a TEGDMA-based polymer fiber battery with high flexibility, retaining 99.4% capacity after 10 000 bending cycles between 0° and 90° (Fig. 10e).

Overall, 1D architectures turn polymer electrolytes from mechanically weak ion conductors into reinforced, stress-dissipating membranes that better resist crack growth and dendrite penetration.

Taken together, the three mechanisms act on complementary aspects of electrolyte performance. Structure-directed transport primarily governs bulk ionic conductivity by reducing pathway tortuosity, interfacial regulation governs salt dissociation and local Li^+^ availability by extending functional interfaces, and stress transfer governs mechanical durability by forming continuous load-bearing networks. A single 1D architecture often engages more than one of these mechanisms at once, and it is their combination that underlies the overall performance gains.

## 5 1D architectures under practical operating conditions

Reported estimates suggest that next-generation EV solid-state batteries require electrodes with a practical areal capacity of at least 7 mAh cm^−2^ and a rate capability of at least 4C to meet long-range operation requirements.^128^ Polymer electrolyte-based thin-film batteries are important for miniaturized electronics and Internet of Things devices. For practical use, they are expected to achieve an areal capacity of at least 3 mAh cm^−2^ and support fast charge–discharge operation, such as rates above 20C.^129^ Current polymer electrolytes still fall short of these practical targets, especially under demanding operating conditions.^130^ In this context, 1D architectures offer a possible route to narrow the gap between laboratory performance and practical application.

### 5.1 Cathodes with high mass loading

Cathodes with high mass loading amplify the limitations associated with ion supply and nonuniform reaction distribution in the thickness direction.^131^ As electrodes become thicker, conventional polymer electrolytes often fail to maintain uniform ion supply.^132–134^ This causes insufficient active material utilization within the electrode and limits both specific capacity and rate performance.^135,136^ To address this issue, 1D architectures have been introduced to construct through-thickness ion transport pathways, thereby reducing tortuosity and improving cathode utilization.

**Fig. 11:**
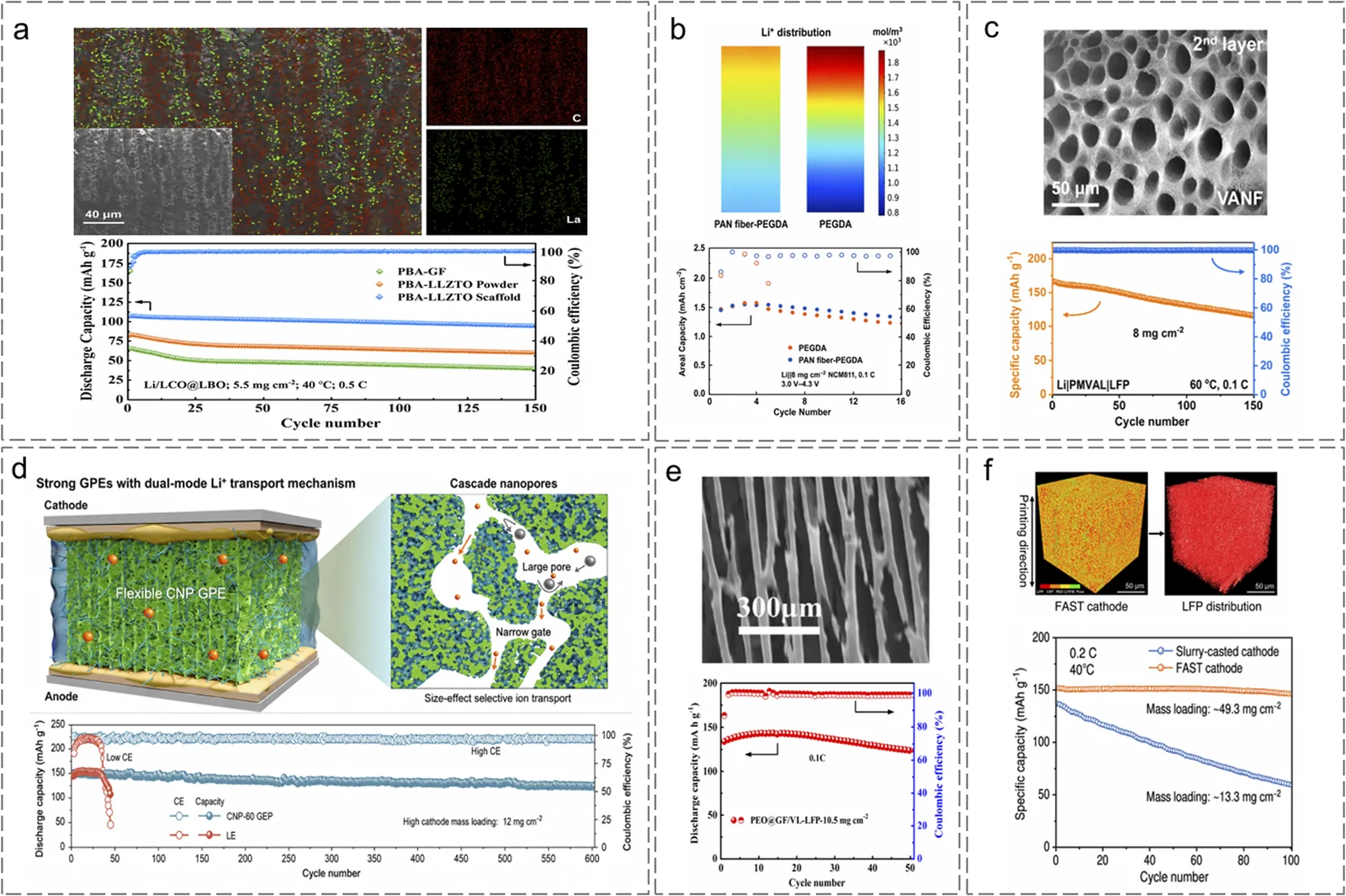
1D architectures for cathodes with high mass loading. (a) Aligned LLZTO scaffold electrolyte for improving Li^+^ supply in high-loading cathodes. Adapted with permission.^137^ Copyright 2024, Elsevier. (b) PAN fiber-PEGDA electrolyte showing uniform Li^+^ distribution and stable NCM811 cycling. Adapted with permission.^138^ Copyright 2025, Wiley-VCH. (c) Vertically porous ANF framework for reducing Li^+^ transport disorder in LFP cells. Adapted with permission.^139^ Copyright 2025, Wiley-VCH. (d) CNP-60 GPE with cascade nanopores for Li^+^-selective transport and LFP cycling at high mass loading. Adapted with permission.^140^ Copyright 2026, Wiley-VCH. (e) Freeze-cast VL-LFP cathode with shortened through-thickness Li^+^ transport distance. Adapted with permission.^141^ Copyright 2019, Elsevier. (f) 3D-printed CNT channels for low tortuosity Li^+^ transport in FAST cathodes with ultrahigh loading. Adapted with permission.^142^ Copyright 2025, AAAS.

Electrolyte-side 1D scaffolds provide a direct route to strengthen Li^+^ supply toward high-loading cathodes. Chen *et al.*^137^ developed a PBA-based composite polymer electrolyte with vertically aligned LLZTO scaffolds. The continuous La distribution in Fig. 11a confirms the formation of connected ceramic pathways across the electrolyte. At an LCO@LBO loading of 5.5 mg cm^−2^, the electrolyte retains 87.8% of its capacity after 150 cycles at 0.5C. This concept can be extended from ceramic scaffolds to polymer-based fibrous networks. Zhang *et al.*^138^ developed PAN-fiber-reinforced PEGDA solid polymer electrolyte. The electrolyte shows more uniform Li^+^ distribution and better cycling stability with an 8 mg cm^−2^ NCM811 cathode, indicating improved Li^+^ supply and cathode utilization under increased loading (Fig. 11b). Beyond reinforcing the electrolyte matrix, vertically continuous pores can further reduce through-membrane transport disorder. Da *et al.*^139^ constructed an ANF framework with vertical pores, followed by *in situ* polymerization of PEGMEA and LiTFSI. The through-membrane channels reduced Li^+^ transport disorder. As a result, the Li‖LFP cell retained 70% of its capacity after 150 cycles at a high LFP loading of 8.0 mg cm^−2^ (Fig. 11c). For higher-loading LFP cathodes, Lv *et al.*^140^ used a cellulose-nanofibril-based 1D network to construct CNP-60 GPE. The GPE maintains Li^+^ supply through cascade nanopores that combine fast ion diffusion with Li^+^-selective transport. The Li‖LFP cell with a loading of 12 mg cm^−2^ retained 87% capacity after 600 cycles (Fig. 11d). Yang *et al.*^141^ used freeze casting to construct vertically aligned VL-LFP composite cathodes. This structure converted the long-distance, top-to-bottom Li^+^ transport in conventional thick electrodes into bidirectional short-distance transport along vertical lamellae. As a result, the Li^+^ transport distance decreased from about 60 µm to about 10 µm. The cathode delivered an areal capacity of 1.52 mAh cm^−2^ (Fig. 11e). Cao *et al.*^142^ used 3D printing to vertically align CNTs in a composite cathode and then infiltrated PEO–LiTFSI. The ionic tortuosity factor decreased from 4.7 to 1.5, and nano-CT showed low-tortuosity aligned channels in the FAST cathode. At a high LFP loading of 49.3 mg cm^−2^, the cathode retained 148.2 mAh g^−1^ after 100 cycles at 0.2C (Fig. 11f). Representative 1D architectures used for cathodes with high mass loading are summarized in Table 5. Most 1D-architecture systems to date have been demonstrated with LFP cathodes, and their behavior with high-voltage cathodes such as NCM811 or LCO is less explored. This points to a design consideration worth further study: the continuous interfaces that lower tortuosity and accelerate Li^+^ transport also define the electrolyte/cathode contact, so their chemical stability becomes increasingly important as the operating voltage rises. Encouragingly, the tunable nature of 1D architectures offers a direct route to address this, for example by tailoring the interfacial phase with a fluorinated or otherwise oxidation-resistant sheath, so that the same continuity that benefits transport can also support stable operation at high voltage.

**Table 5:** 1D architectures for cathodes with high mass loading

Structure	Polymer matrix	Cathode loading	Performance	Ref.
LLZTO scaffolds	PBA	LCO@LBO	87.8% capacity retention after 150 cycles at 0.5C	137
		5.5 mg cm^−2^		
PAN fiber	PAN/PEGDA	NCM811	Maintained areal capacity of 1.4 mAh cm^−2^ after 16 cycles	138
		8 mg cm^−2^		
ANFs	PEGMEA	LFP	70% capacity retention after 150 cycles at 0.1C	139
		8.0 mg cm^−2^		
CNP	PIL	LFP	87% capacity after 600 cycles	140
		12 mg cm^−2^		
VL-LFP	PEO	LFP	87.6% capacity retention after 50 cycles at 0.1C	141
		10.5 mg cm^−2^		
CNT channels	PEO	LFP	92.2% capacity retention after 100 cycles at 0.2C	142
		49.3 mg cm^−2^		

### 5.2 High-rate and high-current operation

Unlike cathodes with high mass loading, where insufficient reactions in deep electrode regions are the main issue, high-rate and high-current operation is mainly limited by insufficient instantaneous Li^+^ supply.^143^ Conventional polymer electrolytes often fail to maintain the interfacial Li^+^ concentration in time. This can lead to local current concentration and concentration polarization.^144^ In this context, 1D architectures provide an effective way to maintain Li^+^ transport and stabilize the interfacial ion distribution under demanding operating conditions. Representative 1D architectures for fast cycling and high-current operation are summarized in Table 6.

**Table 6:** 1D architectures for fast cycling and high-current operation in polymer electrolytes

Structure	Polymer matrix	Battery performance	Ref.
Ordered COF channels	PVDF-HFP	80% capacity retention after 250 cycles at 5C	145
Nanocellulose bundle channels	PVDF-HFP	86% capacity retention after 100 cycles at 5C	146
PDA@LLTO fibers	PEGMA/PEGDE	64.2% capacity retention after 1000 cycles at 5C	147
Ta-doped LaOOH chains	PEO	90% capacity retention after 200 cycles at 3C	148
PVDF-HFP fibers	PVDF-HFP	90% capacity retention after 500 cycles at 10C	149
CGVA	PEO	Li stable for 200 h at 1 mA cm^−2^ and 0.5 mAh cm^−2^	150
Ag NWs	DOL/ECH	Li stable for 200 h at 1 mA cm^−2^	151
UiO-66-NH_2_/F-PI	PVDF-HFP	Li stable for 1800 h at 1 mA cm^−2^ and 1 mAh cm^−2^	152
PI fibrous skeleton	PPT	Li stable for 1600 h at 0.5 mA cm^−2^ and 0.5 mAh cm^−2^	153

#### 5.2.1 High-rate operation

High-rate cycling requires not only high ionic conductivity, but also fast and uniform Li^+^ flux under strong concentration gradients. In this context, 1D architectures improve high-rate performance by converting disordered ion migration into spatially regulated transport, increasing Li^+^ selectivity, and suppressing concentration polarization.

**Fig. 12:**
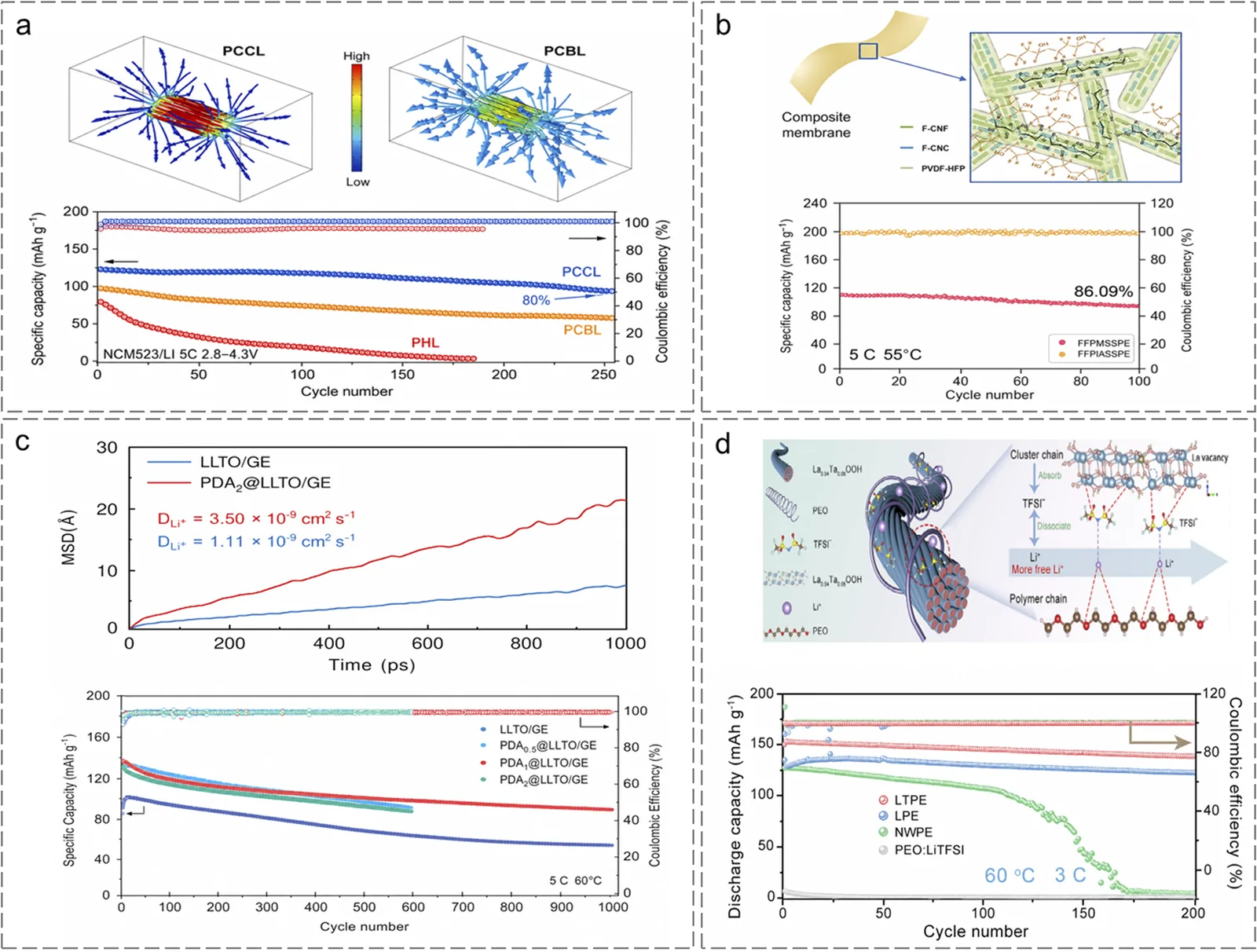
1D architectures for high-rate cycling in polymer electrolytes. (a) PCCL with ordered COF nanochannels for directional Li^+^ flux and 5C cycling. Adapted with permission.^145^ Copyright 2026, Oxford University Press. (b) Bioinspired nanocellulose bundle channels for 5C cycling. Adapted with permission.^146^ Copyright 2026, Springer Nature. (c) PDA-modified LLTO fibers for enhanced Li^+^ diffusion and long-term 5C cycling. Adapted with permission.^147^ Copyright 2026, Springer Nature. (d) Ta-doped LaOOH cluster chains for interfacial Li^+^ transport and 3C cycling. Adapted with permission.^148^ Copyright 2025, Wiley-VCH.

For example, Nie *et al.*^145^ showed that PCCL provides ordered 1D COF nanochannels for directional Li^+^ transport. The simulated Li^+^ flux is more concentrated in PCCL than in PCBL, and the Li‖NCM523 cell retains 80% capacity after 250 cycles at 5C, confirming that continuous nanochannels help sustain cycling stability under high-rate operation (Fig. 12a). Gao *et al.*^146^ further demonstrated this principle using a bioinspired 1D nanocellulose bundle network to construct FFP/ASSPE. The continuous fibrous channels guide Li^+^ transport and reduce migration disorder, enabling the Li‖LFP cell to retain 86.09% capacity after 100 cycles at 5C (Fig. 12b). 1D architectures can also use their extended surfaces to impose ion selectivity along the migration pathway. Li *et al.*^147^ combined PDA@LLTO with polyether matrix. The –OH and –NH groups on the surface of LLTO fibers immobilized TFSI^−^ through hydrogen bonding, increasing the Li^+^ transference number to 0.68 and suppressing concentration polarization. Meanwhile, more free Li^+^ was generated, and the Li^+^ diffusion coefficient increased by 3.2 times. As a result, the Li‖LiFePO_4_ cell still retained a capacity of 80.7 mAh g^−1^ after 1000 cycles at 5C (Fig. 12c). A similar effect was observed in chain-like and fibrous ion-conducting systems. Song *et al.*^148^ used Ta-doped LaOOH subnanometer cluster chains in PEO matrix. The chain structure formed continuous interfacial channels with PEO chains, increasing the Li^+^ transference number to 0.62 and reducing concentration polarization at high rates. The Li‖LFP cell retained about 90% capacity after 200 cycles at 3C (Fig. 12d). Hu *et al.*^149^ further extended this strategy by constructing continuous gel ion channels with Debs-Li and PVDF-HFP fibers. The electrolyte showed a Li^+^ transference number of 0.62 and 90.0% capacity retention after 500 cycles at 10C in Li‖LFP cells.

#### 5.2.2 High-current operation

**Fig. 13:**
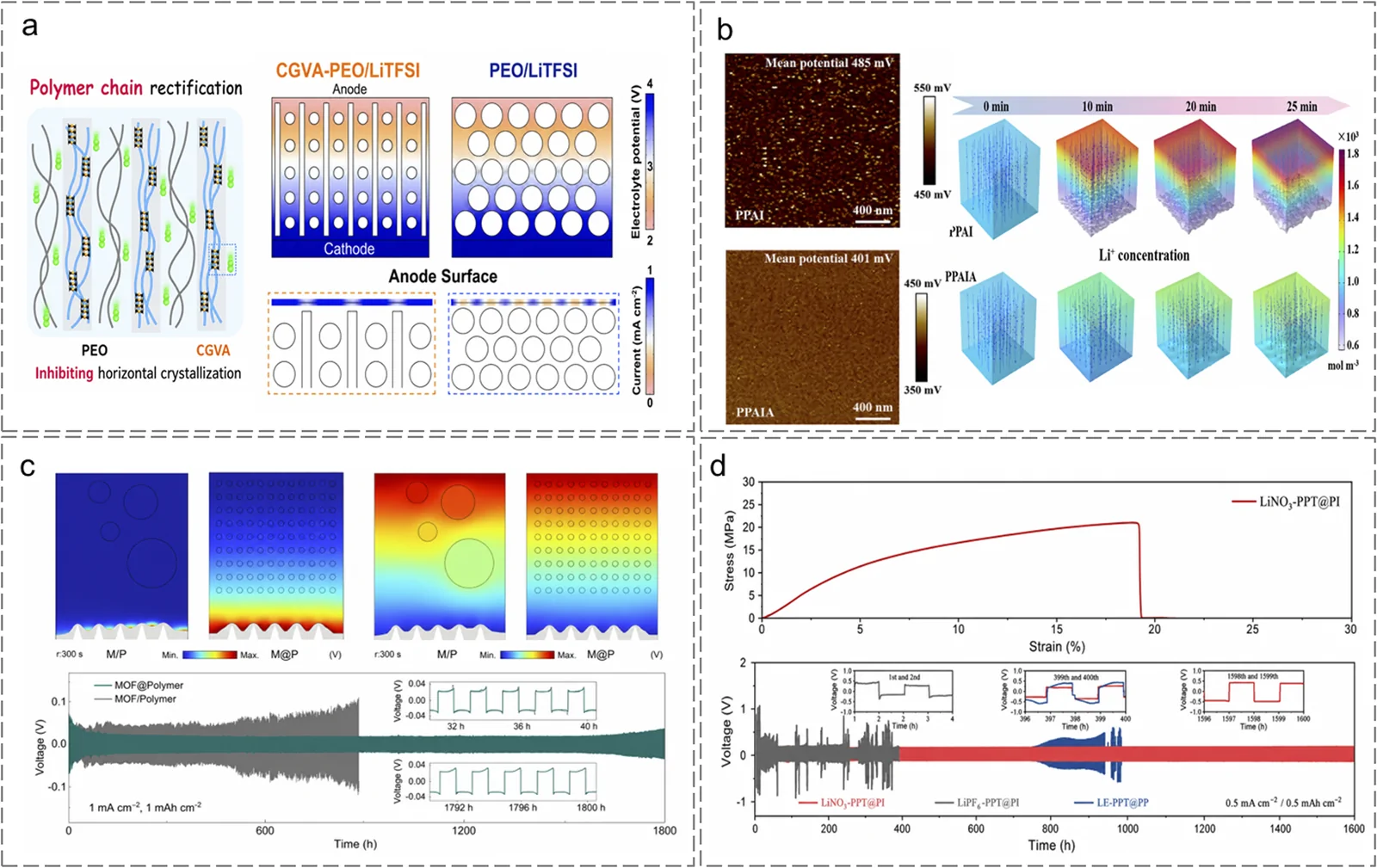
1D architectures for high-current operation in polymer electrolytes. (a) Vertically aligned CGVA arrays for homogenized Li^+^ flux. Adapted with permission.^150^ Copyright 2024, Elsevier. (b) Ag nanowire interlock interface for uniform Li nucleation. Adapted with permission.^151^ Copyright 2026, Wiley-VCH. (c) UiO-66-NH_2_/F-PI nanofiber framework for microdomain consistency. Adapted with permission.^152^ Copyright 2025, Wiley-VCH. (d) PI reinforced LiNO_3_-PPT@PI for stable Li plating and stripping. Adapted with permission.^153^ Copyright 2024, Elsevier.

Under high-current operation, the major challenge is not only rapid Li^+^ transport but also the suppression of local current hotspots and uneven Li deposition. Vertically aligned 1D architectures address this issue by directing Li^+^ transport in the through-plane direction and homogenizing interfacial ion flux. Li *et al.*^150^ accelerated Li^+^ migration by constructing vertically aligned CGVA. This structure reduced ion transport crosstalk and interfacial ion flux disturbance, enabling fast and uniform Li^+^ transport. Finite element simulations showed that CGVA–PEO/LiTFSI had a more uniform current distribution and weaker local concentration polarization. As a result, the critical current density of the polymer electrolyte increased to 2.5 mA cm^−2^. The Li‖Li symmetric cell operated stably for more than 200 h at 1 mA cm^−2^ and 0.5 mAh cm^−2^ (Fig. 13a).

Once Li^+^ flux becomes more directional, regulating the local electric field and Li nucleation environment becomes equally important. Li *et al.*^151^ constructed DOL/ECH-based polymer electrolyte loaded with Ag nanowires. The Kelvin probe force microscopy and finite element simulations showed that the Ag nanowires dispersed charge accumulation near Li nucleation tips and homogenized the local potential and Li^+^ concentration. This interfacial regulation alleviated local current concentration and dendrite growth under high-current operation, enabling the Li‖Li cell to operate stably for 200 h at 1 mA cm^−2^ (Fig. 13b). A similar need for spatial uniformity also appears inside the polymer electrolyte. Feng *et al.*^152^ used an F-PI nanofiber framework to spatially distribute UiO-66-NH_2_ microdomains within the polymer electrolyte. This 1D framework improves microdomain consistency, homogenizes Li^+^ concentration and electric field distributions, and enables stable Li‖Li cycling for about 1800 h at 1 mA cm^−2^ and 1 mAh cm^−2^ (Fig. 13c). Besides flux and field regulation, maintaining mechanical integrity is also necessary during cycling. Wang *et al.*^153^ used a robust PI fibrous skeleton to strengthen LiNO_3_-PPT@PI mechanically. The high tensile strength helps maintain interfacial integrity and suppress dendrite-induced failure, enabling stable Li‖Li cycling for 1600 h at 0.5 mA cm^−2^ and 0.5 mAh cm^−2^ (Fig. 13d).

Taken together, these examples show clear progress on each individual metric, enabling high-loading cathodes to cycle stably and sustaining fast transport at high rates. The remaining step is to combine these gains. So far, high areal capacity and high rate have largely been demonstrated in separate systems rather than within one, and most cases still use LFP. Reaching the benchmarks above simultaneously, and extending these designs to high-voltage chemistries, is thus the natural next direction, to which the directional-transport and continuous-interface features of 1D architectures are well suited.

## 6 Summary and outlook

### 6.1 Summary

This review provides a comprehensive overview of recent advances in 1D architectures for polymer electrolytes, encompassing their classification, design variables, fabrication methods, regulatory mechanisms, and engineering relevance. Central to this review is the recognition that 1D architectures should not be narrowly interpreted as discrete nanowires, nanorods, nanotubes, or nanofibers. Rather, their fundamental significance lies in their role as spatially continuous structural units that organize Li^+^ migration across extended length scales through interconnected networks, aligned arrays, and molecularly or supramolecularly ordered channels. Based on this perspective, these architectures are classified according to intrinsic Li^+^ conductivity into Li^+^-conductive and non-Li^+^-conductive categories, and are further categorized by spatial organization into randomly dispersed, continuous-network, and aligned configurations. Their functional roles are governed by four key design variables: geometry and morphology, spatial continuity, orientation, and interfacial configuration, each of which exerts a decisive influence on ion-transport behavior and electrochemical performance.

Different fabrication methods provide distinct routes to these structures, including electrospinning for continuous fibrous frameworks, template-assisted synthesis for predefined channel size and direction, external-field induction for directional alignment, hydrothermal and solvothermal processing for controlled crystal growth and surface chemistry, and self-assembly for molecular ordering without external processing, thereby enabling the rational construction of 1D architectures with tailored structural features. At the mechanistic level, 1D architectures regulate Li^+^ transport in polymer electrolytes through three primary pathways. First, they enhance the directionality of ion migration and reduce pathway tortuosity, thereby lowering the effective ionic resistance. Second, they extend functional interfaces and amplify interfacial effects, including salt dissociation, anion immobilization, and Li^+^-selective migration. Third, they enable more uniform stress transfer within polymer frameworks and improve mechanical reinforcement. These mechanistic advantages translate into tangible benefits under practical battery operating conditions, where 1D architectures have demonstrated the capacity to shorten effective Li^+^ transport pathways, sustain adequate ion supply at elevated rates, and homogenize interfacial flux distribution across the electrode–electrolyte boundary.

Collectively, 1D architectures represent a powerful structural design strategy for connecting morphology, interface, and spatial organization with Li^+^ transport behavior in polymer electrolytes. As the field progresses from conceptual demonstrations toward practical deployment, translating these architectural advantages into scalable, high-performance electrolyte systems will require addressing several interconnected challenges, which are discussed below.

### 6.2 Outlook

**Fig. 14:**
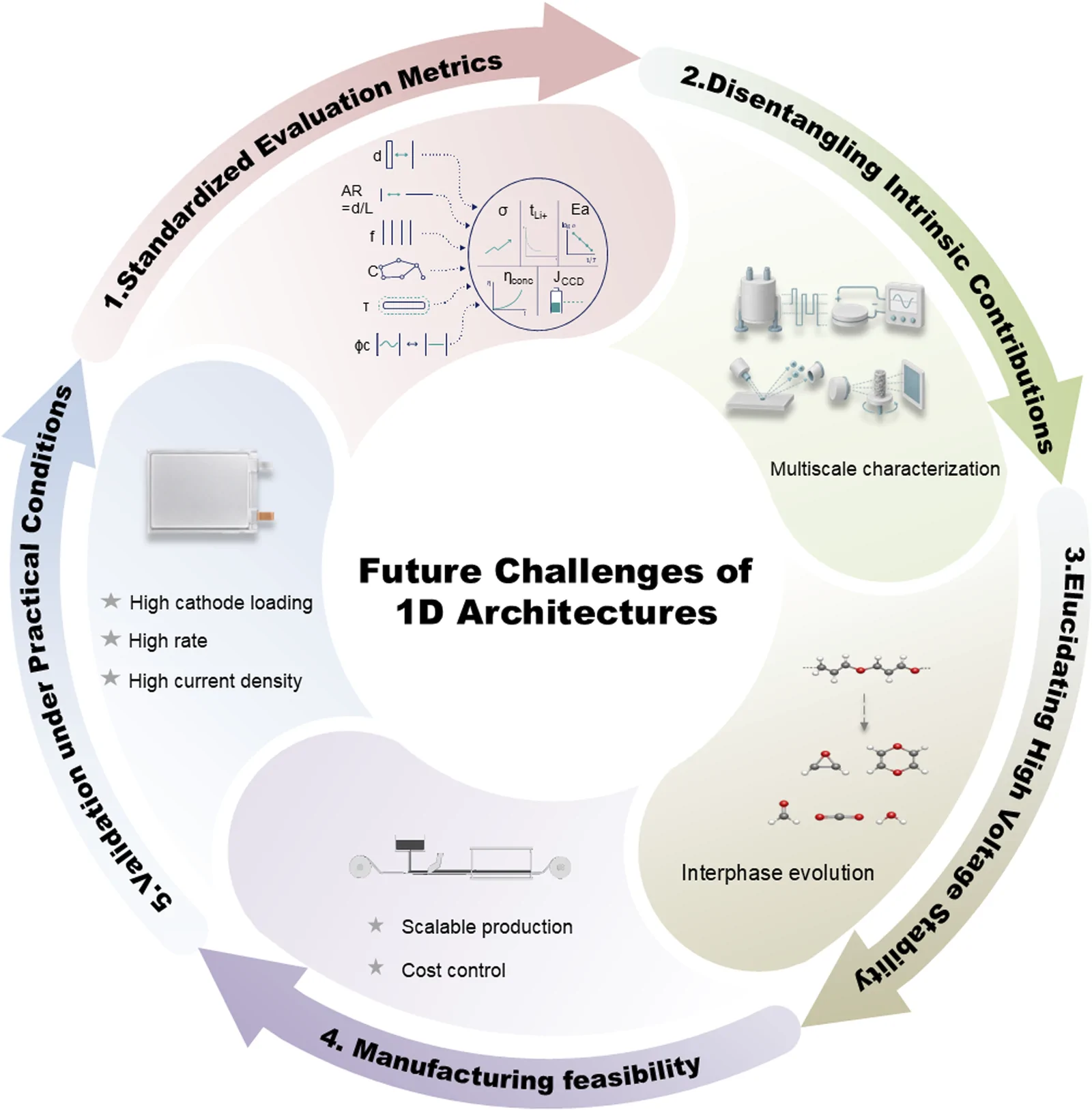
Outlook of 1D architectures in polymer electrolytes.

Despite the considerable promise demonstrated by 1D architectures in polymer electrolytes, their practical realization hinges on overcoming five interrelated challenges that span characterization, mechanistic understanding, stability, manufacturing, and operational validation (Fig. 14).

(1) Establishing unified structural evaluation standards. A unified structural evaluation standard for 1D architectures is still lacking. Quantitative descriptors, such as diameter, aspect ratio, orientation factor, connectivity, interfacial area, tortuosity, and percolation threshold, should be established. These structural parameters should also be correlated with key performance metrics, including ionic conductivity, Li^+^ transference number, activation energy, concentration polarization, and critical current density.

(2) Isolating the intrinsic contribution of 1D architectures to Li^+^ migration. The actual contribution of 1D architectures to Li^+^ migration remains difficult to isolate. Performance gains may result from several factors rather than from the 1D architectures alone. Future studies should combine PFG-NMR, *in situ* impedance, ToF-SIMS, nano-CT, and multiphysics simulations to identify the specific role of 1D architectures.

(3) Clarifying the role of 1D architectures in high-voltage stability. The role of 1D architectures in high-voltage stability is still not well understood. Yao *et al.*^154^ introduced palygorskite nanowires into a PVDF electrolyte, enabling an NMC111 cell to retain 97% capacity after more than 200 cycles at 0.3C. Xi *et al.*^155^ showed that HAP nanowires suppressed superoxide radical formation on PEO chains and improved high-voltage stability. These studies indicate a possible role of 1D architectures in polymer antioxidation and interface stabilization, but systematic evidence remains insufficient.

(4) Advancing scalable and battery-compatible manufacturing. Manufacturing feasibility is also critical for 1D architectures. Although several methods are available, including electrospinning, template methods, external-field induction, hydrothermal or solvothermal growth, and self-assembly, future designs should focus on scalable strategies that are compatible with battery manufacturing and targeted to specific transport bottlenecks.

(5) Validating performance under realistic operating conditions. 1D architectures require further validation under realistic operating conditions. Current studies are mostly based on idealized systems and still fall short of practical battery requirements. Future efforts should focus on deep electrode reaction uniformity, Li^+^ flux replenishment at high rates, and structural and interfacial stability during long-term cycling.

Overall, 1D architectures should be viewed not as fixed material morphologies, but as structural variables for regulating Li^+^ migration in polymer electrolytes. Their future value lies in enabling quantifiable and predictable structured transport design. These principles are not specific to lithium. Framed in this way, their relevance extends beyond lithium-based systems. They should extend to sodium-ion and potassium-ion polymer electrolytes, as well as lithium–sulfur and anode-free systems. Combined with data-driven and high-throughput design, point toward 1D architectures as a general design language for ion transport in solid-state batteries.

## Author contributions

Tian Wang: conceptualization, methodology, investigation, data curation, formal analysis, visualization, writing – original draft, and writing – review and editing. Xinyang Li: conceptualization, investigation, visualization, and writing – review and editing. Dan He: writing – review and editing. Shujiang Ding: conceptualization, supervision, project administration, funding acquisition, and writing – review and editing. Na Li: conceptualization, supervision, project administration, funding acquisition, and writing – review and editing.

## Conflicts of interest

There are no conflicts to declare.

## Data Availability

No primary research results, software or code have been included and no new data were generated or analysed as part of this review.
